# Supplementary material for: Impact of perioperative organ injury on morbidity and mortality in 28 million surgical patients
Source: Nat Commun. 2025 Apr 9;16:3366. doi: 10.1038/s41467-025-58161-2 (PMC11982547; doi:10.1038/s41467-025-58161-2)
Supplement: Supplementary file 1 — Supplementary Information [file 41467_2025_58161_MOESM1_ESM.docx]

**SUPPLEMENTARY APPENDIX**

**Impact of Perioperative Organ Injury on Morbidity and Mortality**

**in 28 Million Surgical Patients**

Investigators: Felix Kork, Yafen Liang, Adit Ginde, Xiaoyi Yuan, Rolf Rossaint, Alex S. Evers, and Holger K. Eltzschig.

Table of Contents

Supplementary Methods 4

Patients 4

Data source and access 4

Variables and Definitions 6

Outcomes 6

Exposures 6

Confounders 6

Statistical Analysis 7

Supplementary Figures……………………………………………………………………………..9

Figure S1: Comparison of in-hospital mortality and morbidity between patients with perioperative organ injury and without. 9

Figure S2: Comparison of in-hospital mortality and morbidity between patients with perioperative delirium and without. 10

Figure S3: Comparison of in-hospital mortality and morbidity between patients with perioperative stroke and without. 11

Figure S4: Comparison of in-hospital mortality and morbidity between patients with perioperative acute myocardial infarction and without. 12

Figure S5: Comparison of in-hospital mortality and morbidity between patients with perioperative acute respiratory distress syndrome and without. 13

Figure S6: Comparison of in-hospital mortality and morbidity between patients with perioperative pulmonary embolism and without. 14

Figure S7: Comparison of in-hospital mortality and morbidity between patients with perioperative liver injury and without………………………………………………………..……………………15

Figure S8: Comparison of in-hospital mortality and morbidity between patients with perioperative acute kidney injury and without…………………………………………………………………….16

Figure S9: Contribution of different types of organ injury to perioperative death determined by attributable fraction.. 17

Figure S10: Perioperative organ injury projected as one of the leading causes of death in Germany and the United States in 2017. 18

Supplementary Tables 19

Table S1: Characteristics of the study cohort categorized by perioperative death. 19

Table S2: Perioperative organ injury, in-hospital mortality and HLOS of 28,350,953 surgical patients categorized by type of surgery…………………………………………………….……….21

Table S3: Types and subtypes of perioperative organ injury in 28,350,953 hospitalized surgical patients. 22

Table S4: Four models in 28,350,953 patients describing the association of any perioperative organ injury with in-hospital mortality and morbidity. 23

Table S5: Comparison of perioperative outcome between patients with no organ injury and different numbers of organ injury. 24

Table S6: Four models in 28,350,953 patients describing the association of multiple perioperative organ injuries with in-hospital mortality and morbidity. 25

Table S7: Four models in 28,350,953 patients describing the association of individual perioperative organ injuries with in-hospital mortality and morbidity. 26

Table S8: Three sensitivity analysis models in 28,350,953 patients confirming the association of any perioperative organ injury with morbidity 27

Table S9: Three sensitivity analysis models in 28,350,953 patients confirming the association of the number of perioperative organ injuries with morbidity 28

Table S10: Three sensitivity analysis models in 28,350,953 patients confirming the association of individual perioperative organ injuries with morbidity 29

Table S11: Risk factors of perioperative organ injury 30

Table S12: Taxonomy of the Operationen- und Prozedurenschlüssel (OPS), the German version of the International Classification of Procedures in Medicine (ICPM), showing in particular detail the subsections from Chapter 5 – Operations”.. 31

Table S13: Details of variable transcoding for procedures and diagnoses. 32

Supplementary References 34

# Supplementary Methods

## Patients

We analyzed data from all hospitalized patients in Germany who underwent surgery and who were discharged or died between January 1st, 2014 and December 31st, 2017. The data was analyzed on a hospital-case basis. Patients who underwent surgery more than once during their hospital stay are represented as one case. Patients who underwent multiple surgeries during multiple hospitalizations are represented as one case per hospitalization.

## Data source and access

We analyzed data from the German Diagnoses Related Groups (G-DRG) Statistik (Source: Research Data Center of the Federal Statistical Office and the Statistical Offices of the Länder, G-DRG statistic, survey years 2014 to 2017, DOIs:10.21242/23141.2014.00.00.1.1.0, 10.21242/23141.2015.00.00.1.1.0, 10.21242/23141.2016.00.00.1.1.0 and 10.21242/23141.2017.00.00.1.1.0, own calculations). The German Federal Statistical Office (Statistisches Bundesamt, www.destatis.de) provides the possibility to analyze these data. The Federal Statistical Office is a subordinate authority of the German Federal Ministry of the Interior, Building, and Community. It collects, processes, presents, and analyzes statistical information on the society, economy, environment, and state. Its mission is to provide neutral, objective, and independent statistics.

Since 2003, German hospitals are compensated according to the G-DRG system on a hospital-case basis. Each year, the *Institut für das Entgeltsystem im Krankenhaus* (InEK GmbH, www.g-drg.de) calculates new compensation fees for the DRGs based on differences in treatment expenses associated with, e.g., age and comorbidities. For this reason, every hospital in Germany is required by law to collect and report the following data without patients’ consent for each hospitalized case after discharge: year of birth, sex, place of residence, admission and discharge date, the reason for admission discharge, main and side diagnoses coded in the International Classification of Diseases and Related Health Problems, tenth edition, German Modification (ICD-10-GM),^1^ conducted surgical and other procedures coded in the German version of the International Classification of Procedures in Medicine (ICPM) as established by the World Health Organization (WHO), the Operationen- und Prozedurenschlüssel (OPS),^2^ and treatment expenses. Diagnosis coding has to follow the diagnosis coding guidelines (Kodierrichtlinien) provided by the InEK GmbH on their homepage (<https://www.g-drg.de/aG-DRG-System_2021/Kodierrichtlinien>) and the ICD-10-GM diagnosis codes provided the Federal Institute for Drugs and Medical Devices (Bundesinstitut für Arzneimittel und Medizinprodukte, BfArM; <https://www.dimdi.de/static/de/klassifikationen/icd/icd-10-gm/kode-suche/htmlgm2020/>), a higher federal authority of the Federal Ministry of Health (Bundesgesundheitsministerium, BMG). Hospitals’ diagnosis and procedure coding are regularly monitored by the Medical Review Board of the Statutory Health Insurance Funds (Medizinischer Dienst der Krankenkassen, MDK). Inaccurately coded billings are not compensated/reimbursed.

After completion of the DRG calculations, the data are deidentified and transferred to the Federal Statistical Office. Most of the variables are made available to scientists for analysis. Since we used deidentified data and accessed the DRG statistics via controlled remote data processing, no Institutional Review Board approval was required. The previous studies used a similar data access approach.^3^ As these data are not entirely anonymous, and scientific use has to follow certain data safety restrictions, scientists can only conduct remote data analyses but do not have access to the actual data. For this purpose, the Federal Statistical Office provides researchers with data structure files with the same syntax and variable coding as the original data, but the data have been randomly shuffled to senselessness. We wrote an analysis protocol as a Stata do-file (Stata 14.2 for MacOS, StataCorp, College Station, TX, USA), tested it on the data structure files, and sent it to the Federal Statistical Office, where the analysis was run on the actual data (Stata 15 for Windows, StataCorp, College Station, TX, USA). The Federal Statistical Office reviews the results of the analyses to prevent the deanonymization of individuals with a strict protocol, i.e., results from the remote analyses will be censored in the following cases: counts in table cells and figures are ≤3, analyses or summary statistics are derived from ≤3 values, counts in inner table cells differ from higher-order table cells ≤2, single or individual values including minima and maxima, residuals, quantiles if ranges include ≤3 cases.

## Variables and Definitions

The Federal Statistical Office provided access to all surgical cases within the G-DRG statistic from 2014 to 2017. The Federal Statistical Office predefined surgical cases as cases with at least one procedure code from chapter 5 of the OPS (see Table S9 for the taxonomy of surgical procedures in the OPS). These were inpatient surgery cases when patients stayed at least overnight. Therefore, no day surgery, intervention, or diagnostic procedures are included. We were given access to the variables age, sex, reason for admission, hospital length of stay (HLOS), ICD-10-GM coded diagnoses, OPS-coded procedures, and reason for discharge. We used ICD-10-GM diagnosis codes and OPS procedure codes to create new categorical variables. Table S10 presents a complete list of codes for each newly created variable.

## Outcomes

The primary endpoint in-hospital death is coded as a reason for discharge, and the secondary endpoint, HLOS, is an original variable within the G-DRG Statistik.

## Exposures

Patients were considered to be suffering from organ injury if any ICD-10-GM code indicated the presence of one of the following diseases: Delirium, Stroke, acute myocardial infarction (AMI), adult respiratory distress syndrome (ARDS), pulmonary embolism (PE), liver injury (LI), or acute kidney injury (AKI; Table S10). We chose these organ injuries because they are the most well-defined and retrievable from the database.

## Confounders

We considered age, sex, emergency admission, and patients’ comorbidities and undergoing high-risk surgery as confounders that could affect the exposure and endpoints in this study. Comorbidities were assessed using the items of the Charlson Comorbidity Index(CCI).^4^ The CCI was initially developed in 1987 as a prognostic tool for comorbid conditions that may alter the risk of short-term mortality for patients in longitudinal studies. The presence of each CCI item was abstracted from the ICD-10-GM diagnoses described previously by Quan and colleagues (Table S10).^5^ In an unstructured consensus based on our clinical experience with the existing literature, we agreed on the following high-risk surgeries as possible confounders for both exposure (organ injury) and endpoints (in-hospital death and HLOS): We considered intracranial surgery, thoracic surgery (excluding cardiac surgery), cardiac surgery, abdominal surgery, and transplantation surgery as high-risk procedures.

## Statistical Analysis

Frequencies are reported as absolute numbers and percentages. Continuous variables are reported as median and quartiles because the secondary endpoint HLOS is usually not normally distributed. Categorical variables were compared using the chi-squared-test, and continuous variables using the Mann-Whitney-U-test. Exact testing was used whenever possible. Different regression models were fit to estimate the association of organ injury with in-hospital death and HLOS. For all regression models, the selection of dependent variables was based on clinical relevance: We considered age, sex, emergency admission, CCI items, and high-risk surgery as possible confounders. High-risk surgeries are defined as thoracic surgery (excluding cardiac surgery), cardiac surgery, abdominal surgery, and transplantation. For in-hospital death, binary logistic regression models were fit and cross-validated using the Stata module cvauroc (k=10; robustness measure: area under the receiver operating curve (AUROC)).^6^ To estimate the association of organ injuries with HLOS, robust regression models were fit and cross-validated using the Stata module ‘crossfold’ (k=10; robustness measure: root mean square error (RMSE)).^7^ Due to the right skew of HLOS, we conducted several sensitivity analyses to confirm these results: We fit (1) linear regression models for log-transformed HLOS, (2) competing risk models with in-hospital death as competing risk for HLOS, and (3) proportional odds models for hospital free days 90. When analyzing Kaplan-Meier curves and corresponding Cox proportional hazard models, we considered HLOS as the time to death if we censored patients at in-hospital death, or HLOS as the time to discharge if we censored patients at hospital discharge.

Analysis code was written with Stata 14 for MacOs (StataCorp, College Station, TX, US) by the authors (F.K.), and this code was run on Stata 15 for Windows (StataCorp, College Station, TX, USA) on computers at the German Federal Statistical Office. Figures were created using Prism 9.0.1 for MacOS (GraphPad, San Diego, CA, USA). The probability of a type I error of less than 0.01% was considered statistically significant.

# Supplementary Figures

## Figure S1: Comparison of in-hospital mortality and morbidity between patients with perioperative organ injury and without.

**
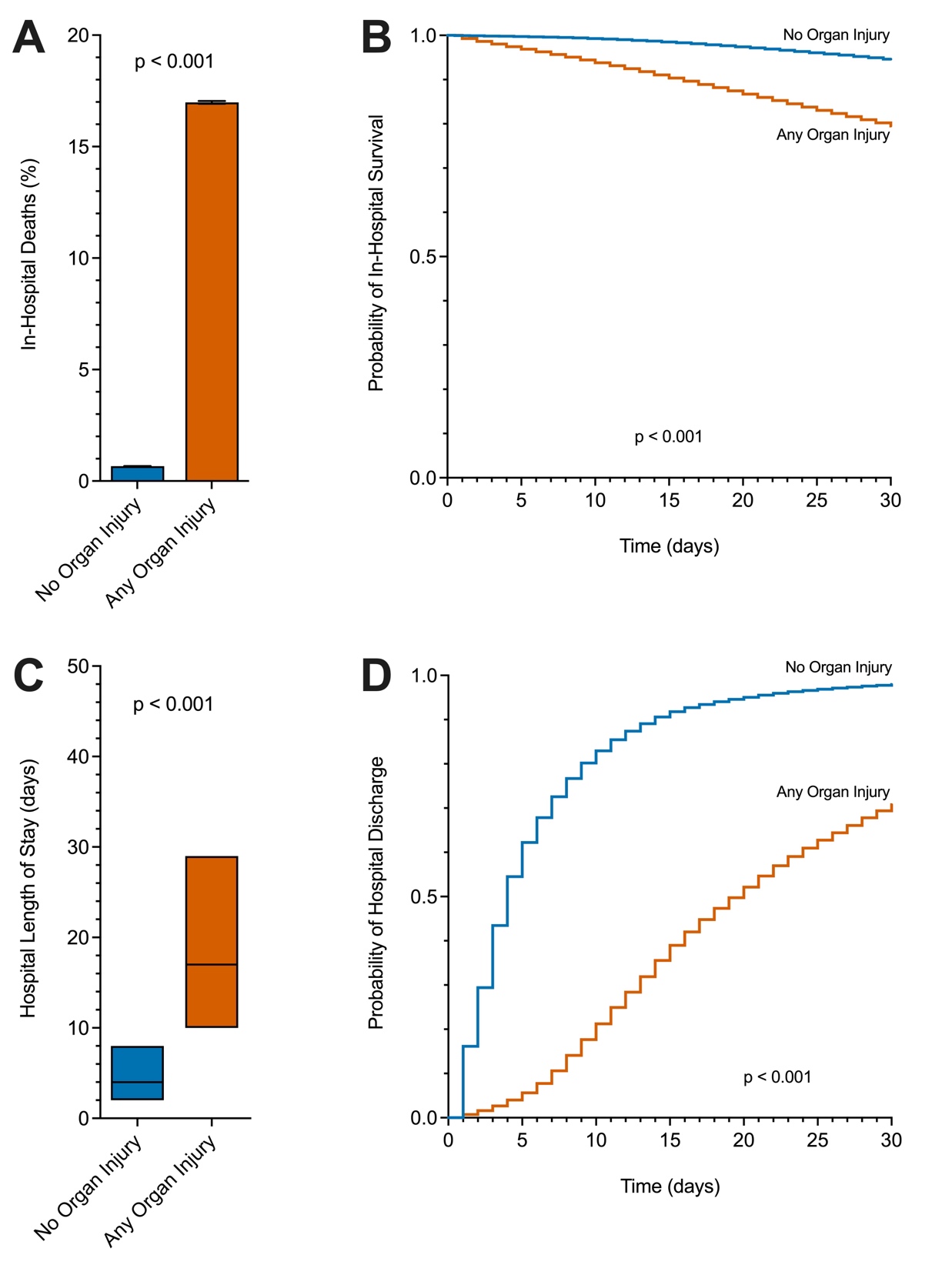
**

**A** Patients who developed perioperative organ injury had higher mortality rates (17.0% vs. 0.7%, chi-squared test, P<0.001) than those who did not (error bars: 95%CI). **B** Kaplan Meier survival curves show the difference in survival between patients with perioperative organ injury and without (log-rank test, P<0.001). **C** Patients who developed perioperative organ injury had longer hospital LOS (17 vs. 4 days, Median test, P<0.001) than patients who did not (median and quartiles). **D** Kaplan-Meier hazard curves for the probability of hospital discharge differ between patients with perioperative organ injury and without (log-rank test, P<0.001).

## Figure S2: Comparison of in-hospital mortality and morbidity between patients with perioperative delirium and without.

**
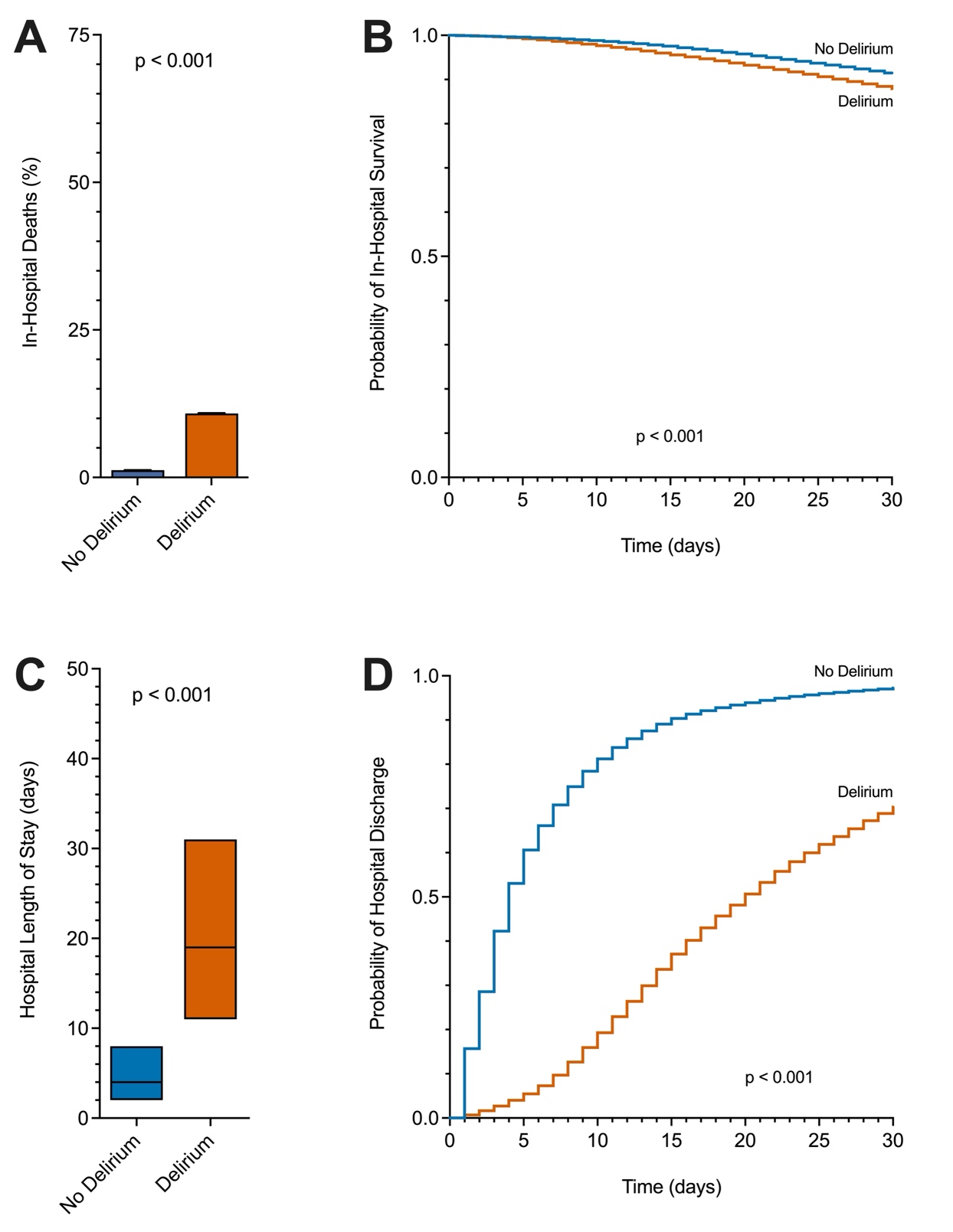
**

**A** Patients who developed perioperative delirium had higher mortality rates (10.8% vs. 1.2%, chi-squared test, P<0.001) than those who did not (error bars: 95%CI). **B** Kaplan Meier survival curves show the difference in survival between patients with perioperative delirium and without (log-rank test, P<0.001). **C** Patients who developed perioperative delirium had longer hospital LOS (19 vs. 4 days, Median test, P<0.001) than those who did not (median and quartiles). **D** Kaplan-Meier hazard curves for the probability of hospital discharge differ between patients with perioperative delirium and without (log-rank test, P<0.001).

## Figure S3: Comparison of in-hospital mortality and morbidity between patients with perioperative stroke and without.

**
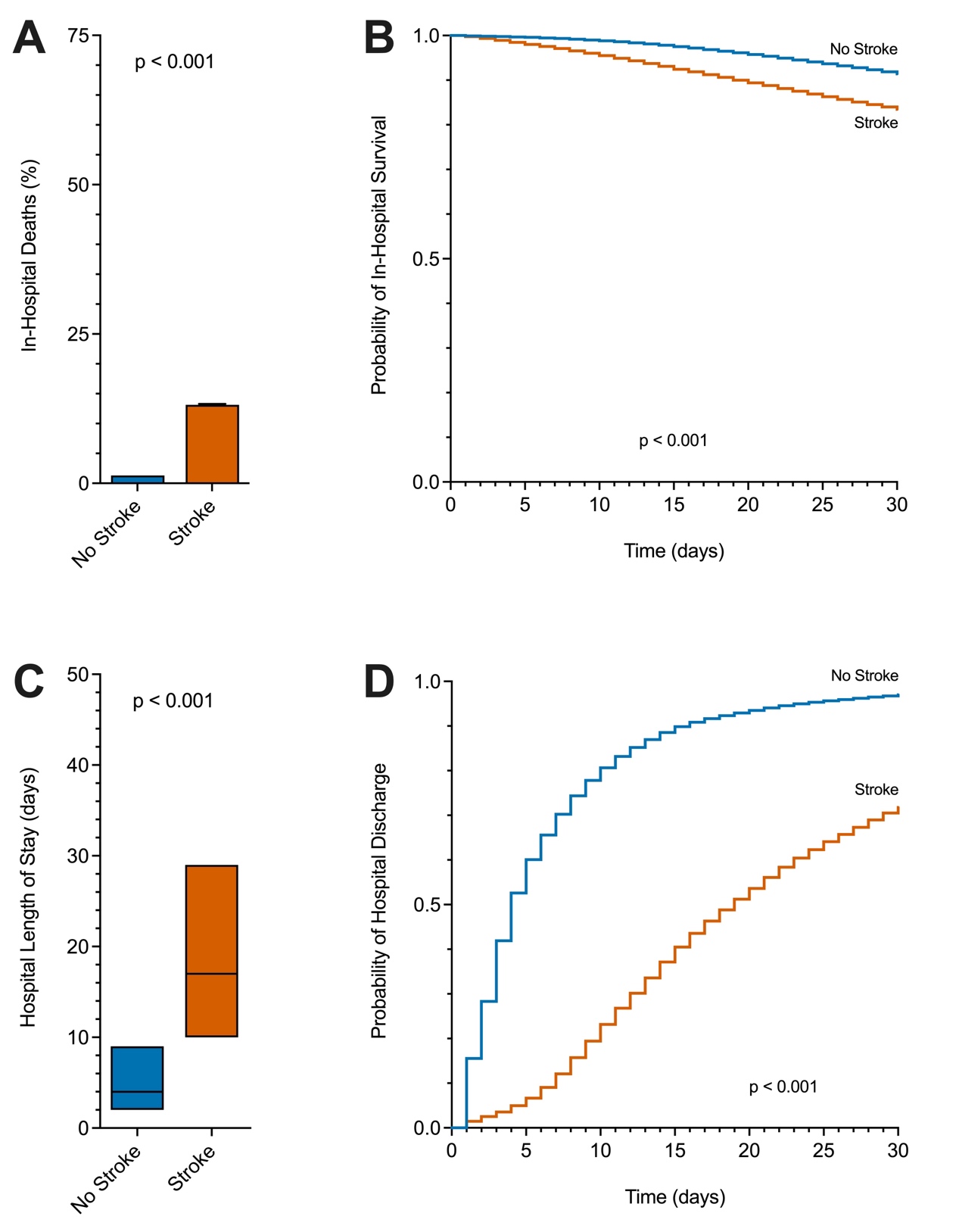
**

**A** Patients who developed perioperative stroke had higher mortality rates (13.1% vs. 1.3%, chi-squared test, P<0.001) than those who did not (error bars: 95%CI). **B** Kaplan Meier survival curves show the difference in survival between patients with perioperative stroke and without (log-rank test, P<0.001). **C** Patients who developed perioperative stroke had longer hospital LOS (17 vs. 4 days, Meidan test, P<0.001) than patients who did not (median and quartiles). **D** Kaplan-Meier hazard curves for the probability of hospital discharge differ between patients with perioperative stroke and without (log-rank test, P<0.001).

## Figure S4: Comparison of in-hospital mortality and morbidity between patients with perioperative acute myocardial infarction and without.


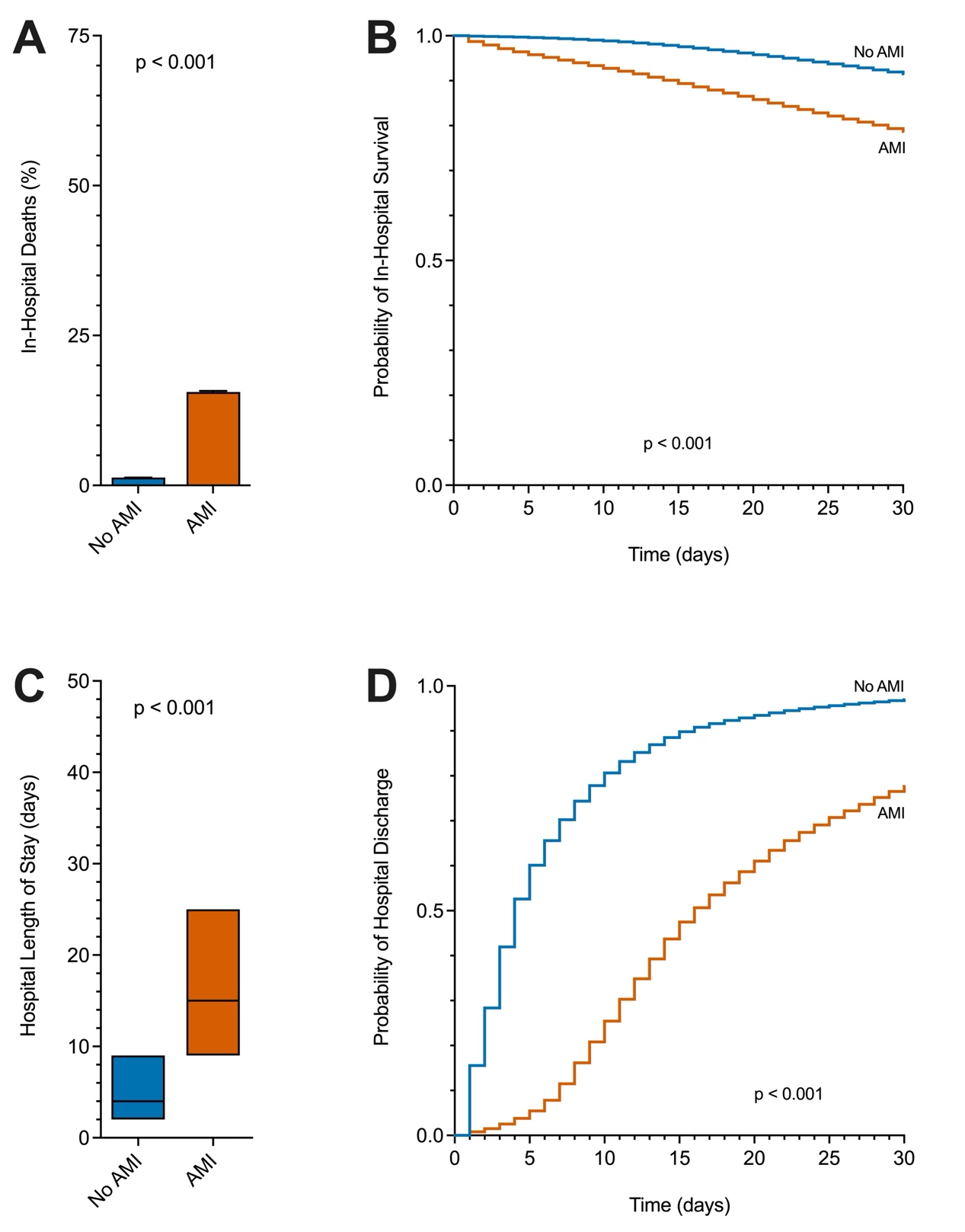


**A** Patients who developed perioperative acute myocardial infarction (AMI) had higher mortality rates (15.6% vs. 1.3%, chi-squared test, P<0.001) compared with patients who did not (error bars: 95%CI). **B** Kaplan Meier survival curves show the difference in survival between patients with perioperative AMI and without (log-rank test, P<0.001). **C** Patients who developed perioperative AMI had longer hospital LOS (15 vs. 4 days, Median test, P<0.001) compared with patients who did not (median and quartiles). **D** Kaplan-Meier hazard curves for the probability of hospital discharge differ between patients with perioperative AMI and without (log-rank test, P<0.001).

## Figure S5: Comparison of in-hospital mortality and morbidity between patients with perioperative acute respiratory distress syndrome and without.


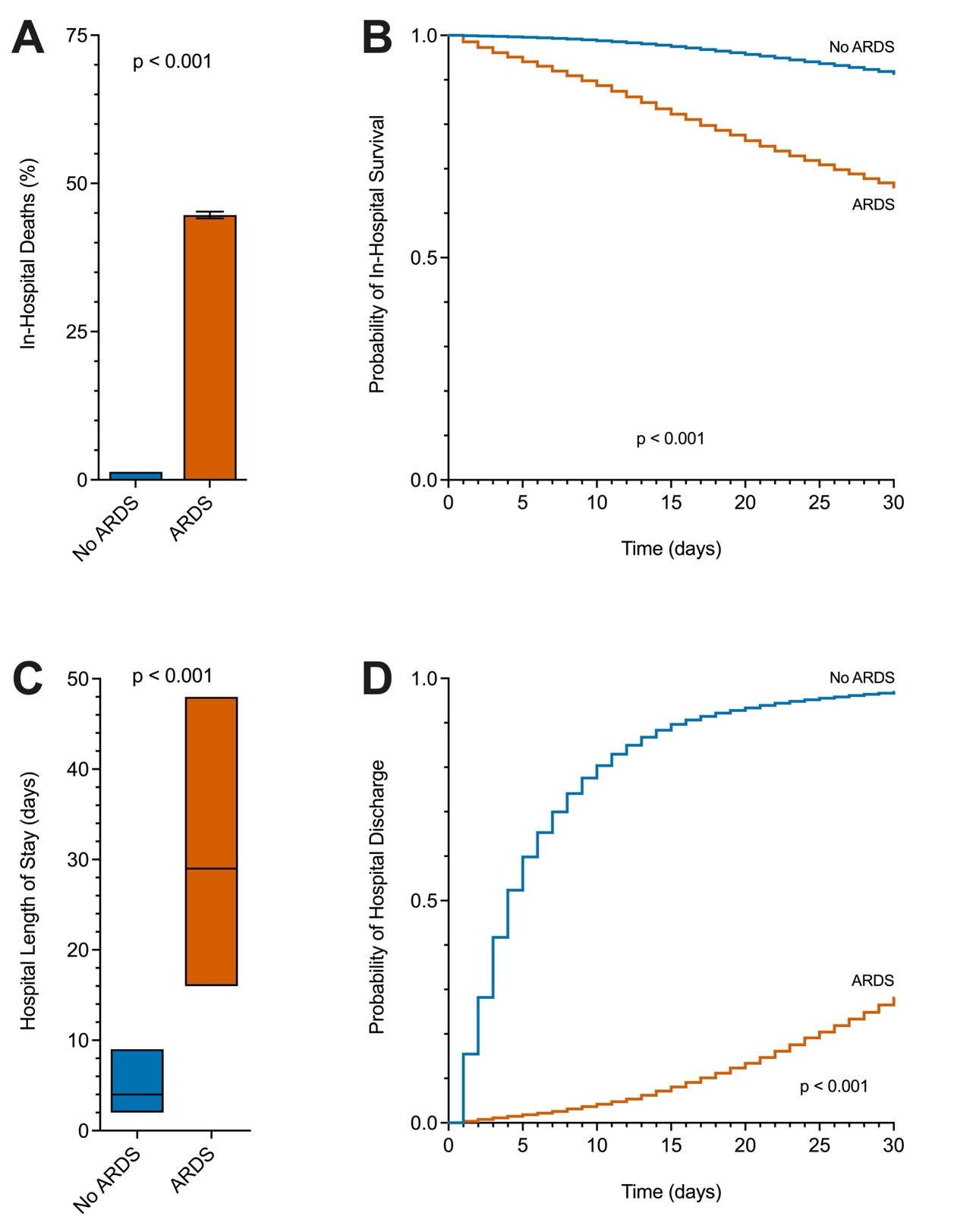


**A** Patients who developed perioperative acute respiratory distress syndrome (ARDS) had higher mortality rates (44.7% vs. 1.3%, chi-squared test, P<0.001) compared with patients who did not (error bars: 95%CI). **B** Kaplan Meier survival curves show the difference in survival between patients with perioperative ARDS and without (log-rank test, P<0.001). **C** Patients who developed perioperative ARDS had longer hospital LOS (29 vs. 4 days, Meidan test, P<0.001) compared with patients who did not (median and quartiles). **D** Kaplan-Meier hazard curves for the probability of hospital discharge differ between patients with perioperative ARDS and without (log-rank test, P<0.001).

## Figure S6: Comparison of in-hospital mortality and morbidity between patients with perioperative pulmonary embolism and without.


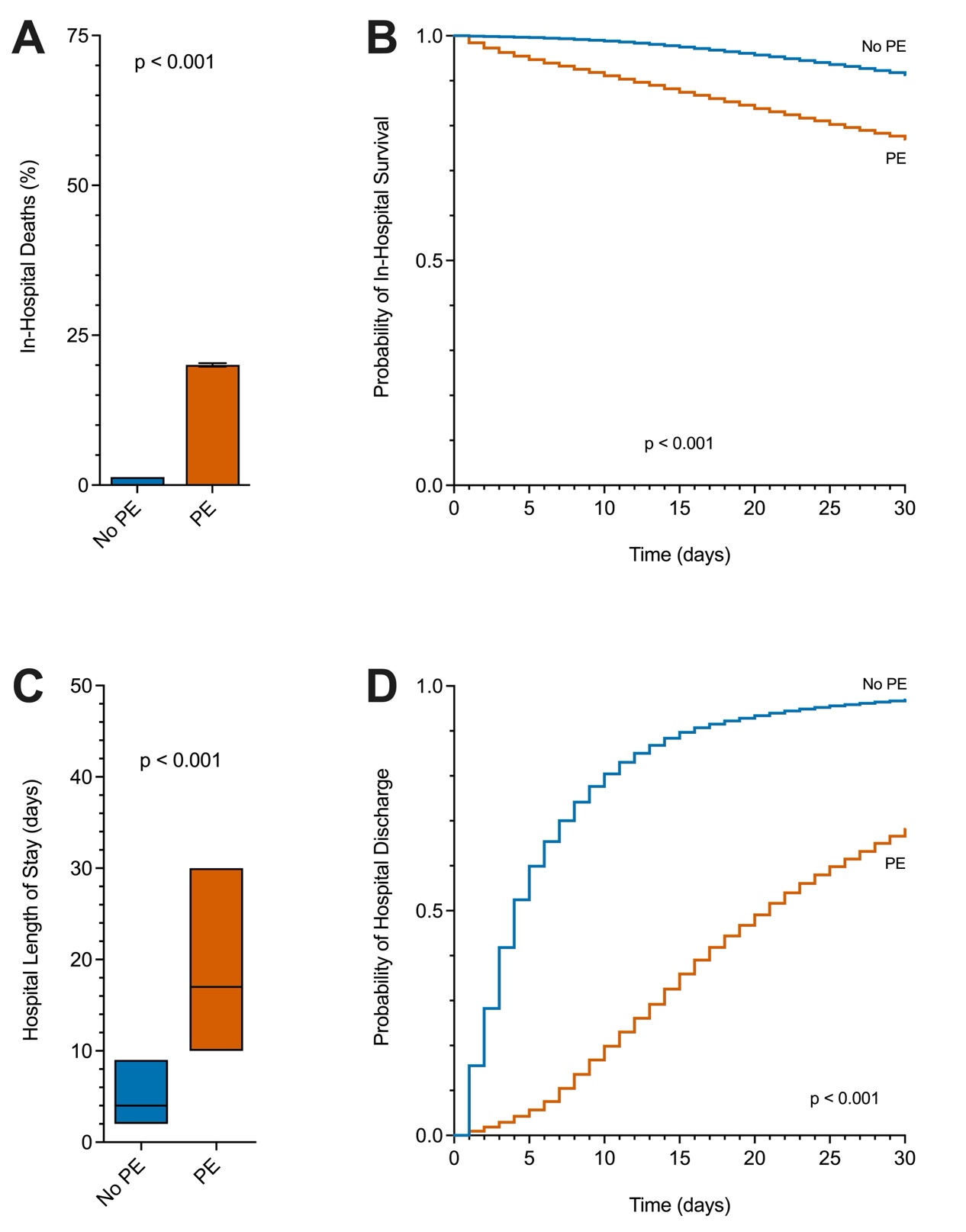


**A** Patients who developed perioperative pulmonary embolism (PE) had higher mortality rates (20.0% vs. 1.3%, chi-squared test, P<0.001) compared with patients who did not (error bars: 95%CI). **B** Kaplan Meier survival curves show the difference in survival between patients with perioperative AKI and without (log-rank test, P<0.001). **C** Patients who developed perioperative AKI had longer hospital LOS (17 vs. 4 days, Meidan test, P<0.001) compared with patients who did not (median and quartiles). **D** Kaplan-Meier hazard curves for the probability of hospital discharge differ between patients with perioperative PE and without (log-rank test, P<0.001).

**Figure S7: Comparison of in-hospital mortality and morbidity between patients with**

**perioperative liver injury and without.**


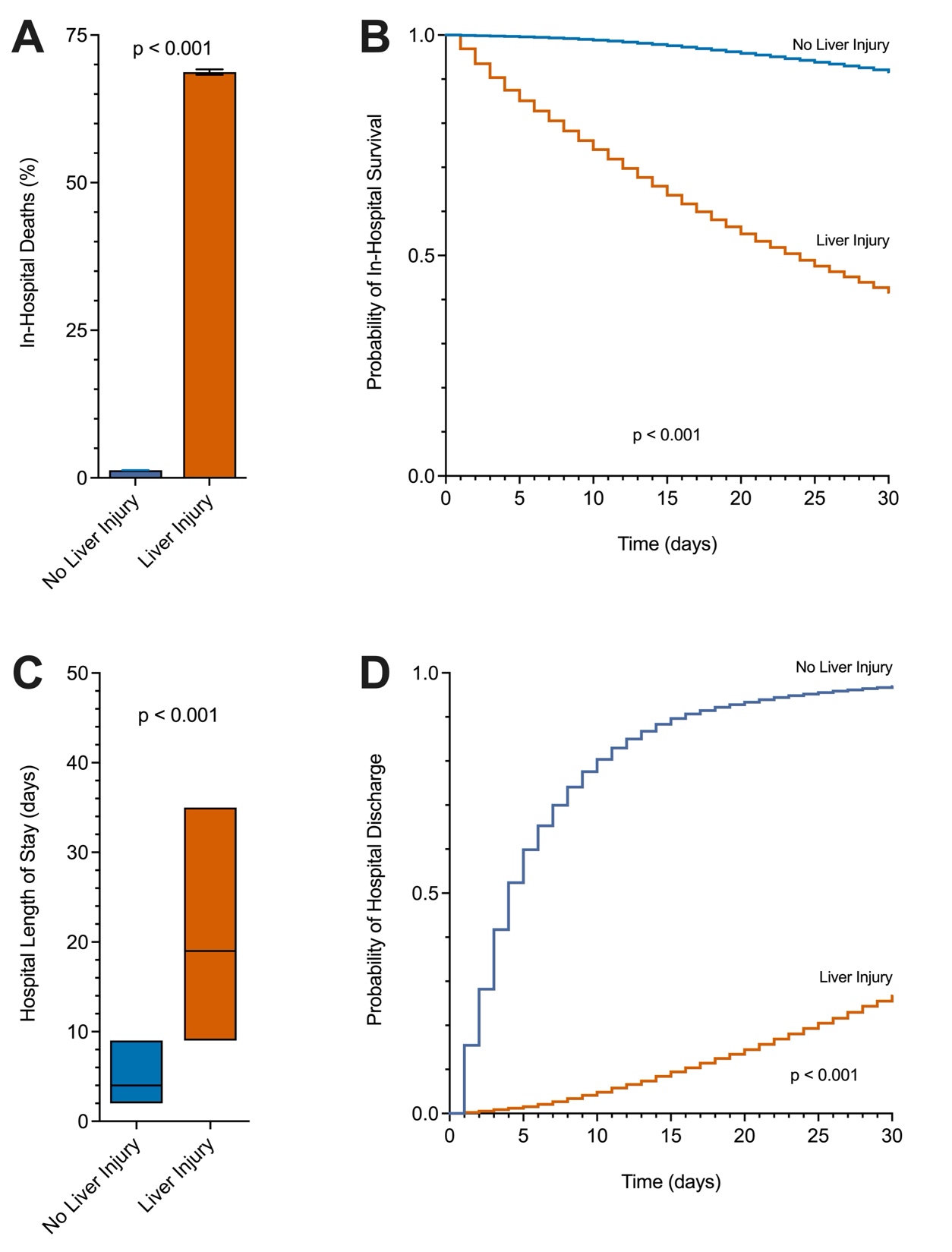


**A** Patients who developed perioperative liver injury (LI) had higher mortality rates (68.7% vs. 1.4%, chi-squared test, P<0.001) compared with patients who did not. Panel (error bars: 95%CI) **B** Kaplan Meier survival curves show the difference in survival between patients with perioperative liver injury and without (log-rank test, P<0.001). **C** Patients who developed perioperative ALI had longer hospital LOS (19 vs. 4 days, Median test, P< 0.001) compared with patients who did not (median and quartiles). **D** Kaplan-Meier hazard curves for the probability of hospital discharge differ between patients with perioperative liver injury and without (log-rank test, P<0.001).

**Figure S8: Comparison of in-hospital mortality and morbidity between patients with perioperative acute kidney injury and without.**


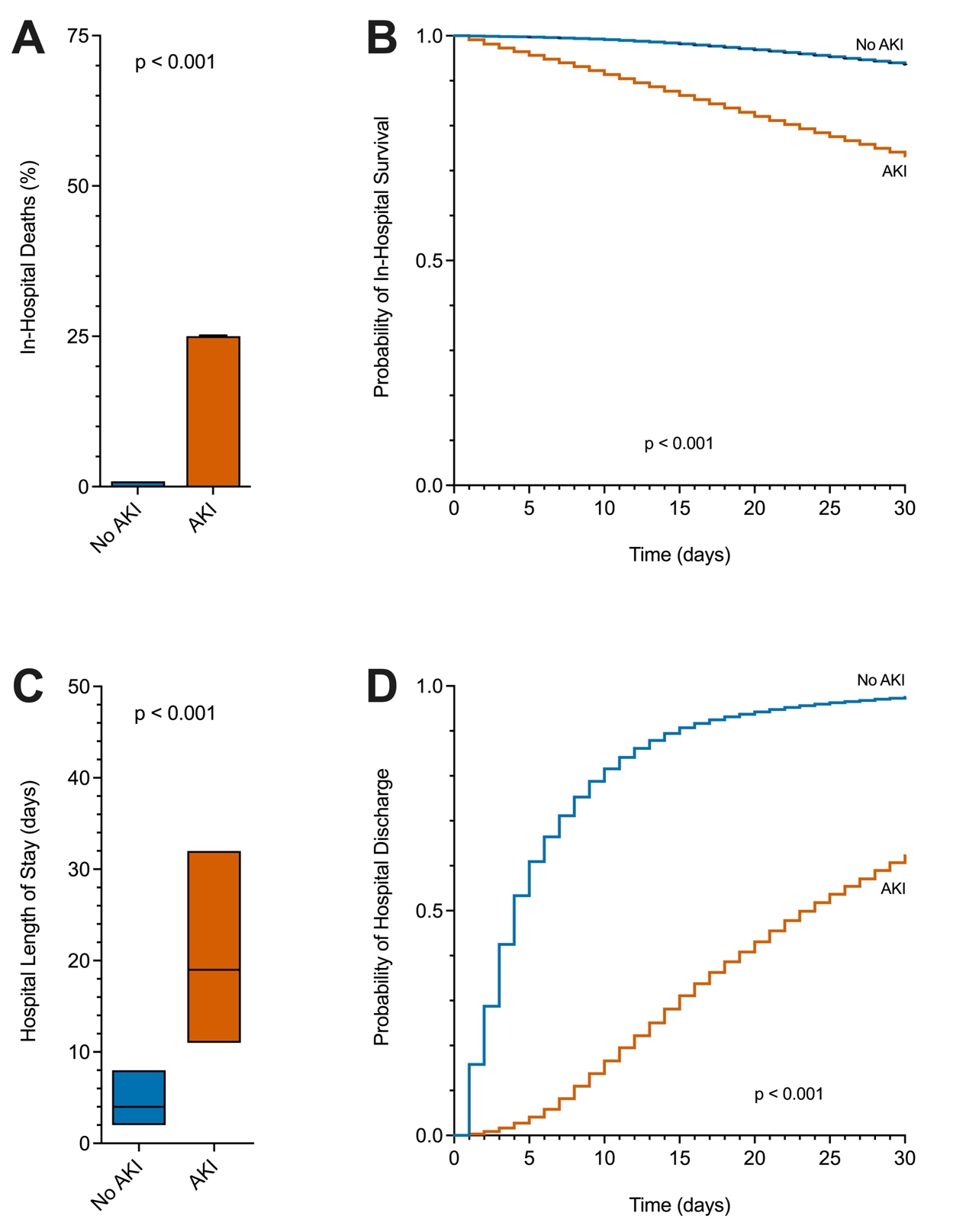


**A** Patients who developed perioperative acute kidney injury (AKI) had higher mortality rates (25.0% vs. 0.9%, chi-squared test, P<0.001) compared with patients who did not (error bars: 95%CI). **B** Kaplan Meier survival curves show the difference in survival between patients with perioperative AKI and without (log-rank test, P<0.001). **C** Patients who developed perioperative AKI had longer hospital LOS (19 vs. 4 days, Median test, P< 0.001) compared with patients who did not (median and quartiles). **D** Kaplan-Meier hazard curves for the probability of hospital discharge differ between patients with perioperative AKI and without (log-rank test, P<0.001).

## Figure S9: Contribution of different types of organ injury to perioperative death determined by attributable fraction.


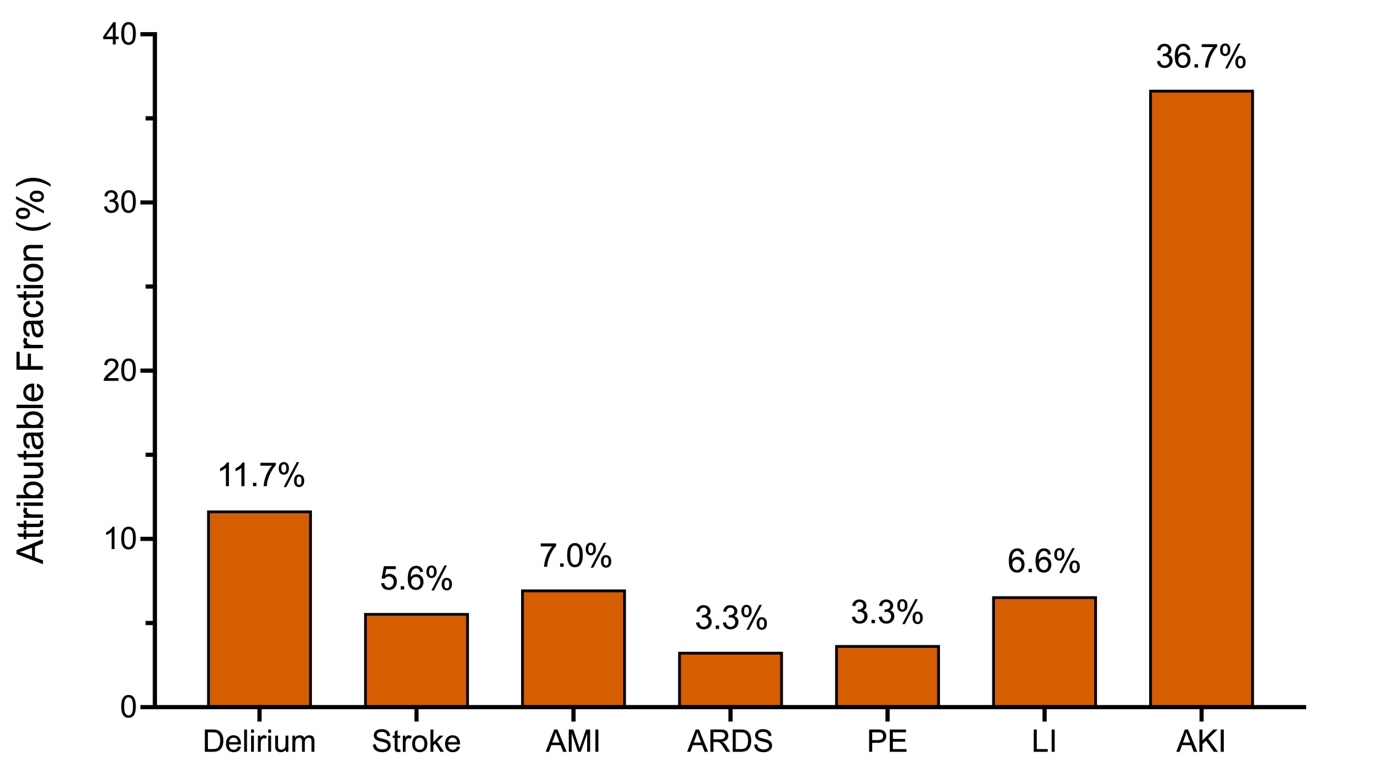


The relative contribution of individual organ injuries to perioperative mortality was determined by calculating the attributable fraction using the following formula: AFp=(Ip-Iu)/Ip, while Ip is the incidence in the population, Iu is the incidence in the unexposed group. AKI is the largest contributor to perioperative death (36.7%) and may serve a priority target for intervention.

## Figure S10: Perioperative organ injury projected as one of the leading causes of death in Germany and the United States in 2017.


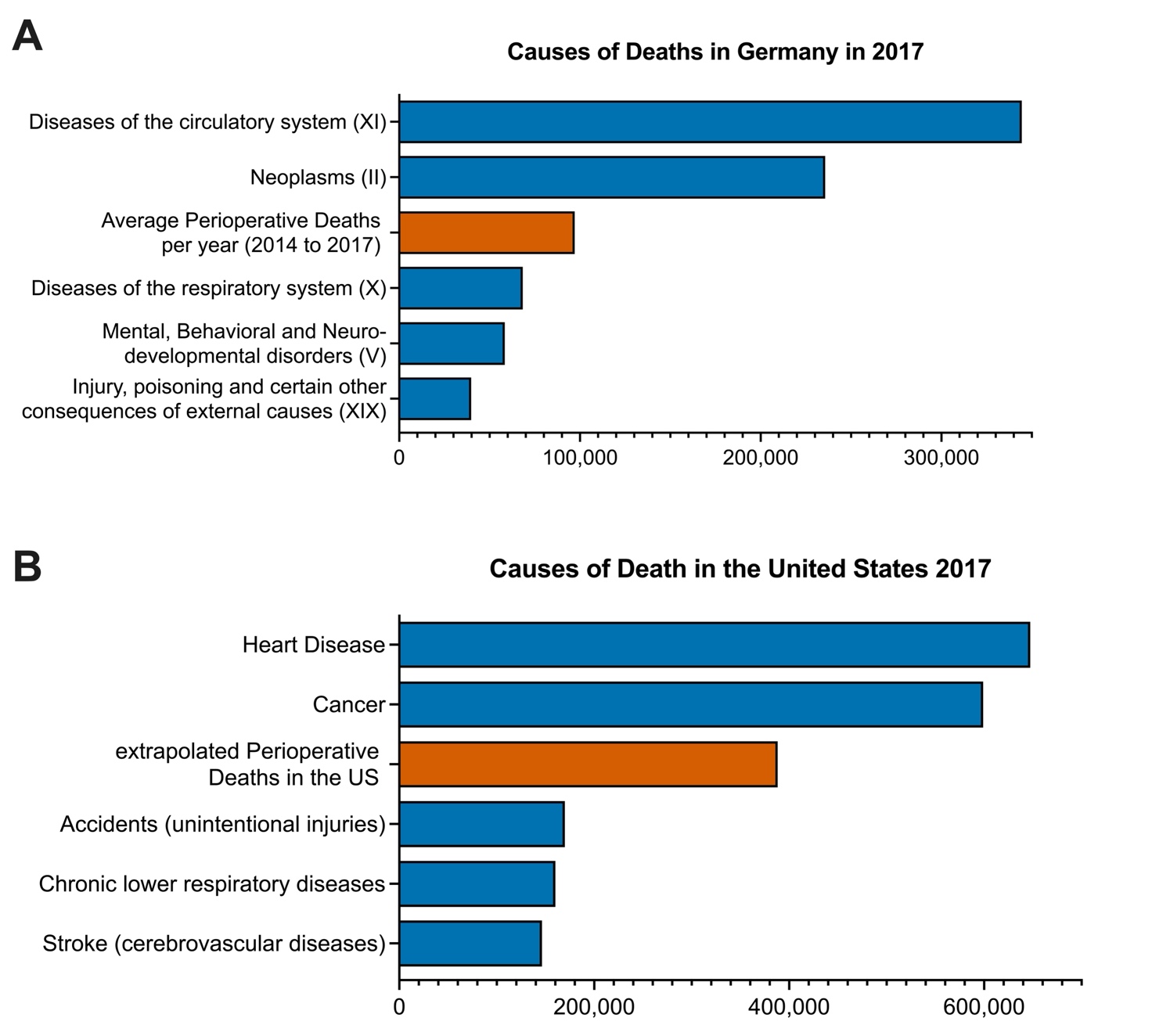


**A** Causes of death in Germany in 2018 (blue) by chapter of the International Classification of Diseases (ICD-10) Chapters according to the German Federal Statistical Office (Statistisches Bundesamt, <https://www.destatis.de/DE/Themen/Gesellschaft> Umwelt/Gesundheit/Todesursachen/Tabellen/gestorbene_anzahl.html, accessed 09 June 2020) and average perioperative deaths (red) including perioperative deaths with organ injury (black).

**B** Causes of Death in the US in 2017 according to the National Center for Health Statistics (NCHS; https://www.cdc.gov/nchs/fastats/deaths.htm, accessed 09 June 2020) with extrapolated perioperative deaths (red) including perioperative deaths with organ injury (black).

#

# Supplementary Tables

## Table S1: Characteristics of the study cohort categorized by perioperative death.

| Characteristics | All patients  (n=28,350,953) | | Deaths  (n=393,157) | | Survivors  (n=27,957,796) | |
| --- | --- | --- | --- | --- | --- | --- |
| Age, median [IQR] | 59 | [39–74] | 77 | [67–84] | 59 | [39–74] |
| Sex, female | 15,390,796 | (54.3%) | 175,016 | (44.5%) | 15,215,780 | (54.4%) |
| Hospital Admission Type |  |  |  |  |  |  |
| Referral from Physician | 19,309,039 | (68.1%) | 103,184 | (26.2%) | 19,205,855 | (68.7%) |
| Emergency | 8,287,300 | (29.2%) | 235,255 | (59.8%) | 8,052,045 | (28.8%) |
| Transfer from another Hospital | 733,057 | (2.6%) | 53,870 | (13.7%) | 679,187 | (2.4%) |
| Transfer from Rehabilitation | 2,188 | (<0.1%) | 117 | (0.02‰) | 2,071 | (<0.1%) |
| Birth | 19,369 | (<0.1%) | 731 | (0.2%) | 18,638 | (<0.1%) |
| Charlson Comorbidity Index, median [IQR] | 0 | [0–2] | 3 | [2–6] | 0 | [0–1] |
| Diabetes Mellitus | 3,843,335 | (13.6%) | 117,156 | (29.8%) | 3,726,179 | (13.3%) |
| Uncomplicated | 2,952,801 | (10.4%) | 76,860 | (19.5%) | 2,875,941 | (10.3%) |
| With end-organ damage | 890,534 | (3.1%) | 40,296 | (10.2%) | 850,238 | (3.0%) |
| Cancer | 3,156,980 | (11.1%) | 112,564 | (28.6%) | 3,044,416 | (10.9%) |
| Non-metastatic | 2,246,180 | (7.9%) | 51,665 | (13.1%) | 2,194,515 | (7.8%) |
| Metastatic | 910,800 | (3.2%) | 60,899 | (15.4%) | 849,901 | (3.0%) |
| Renal Disease | 2,336,360 | (8.2%) | 127,866 | (32.5%) | 2,208,494 | (7.9%) |
| Congestive Heart Failure | 1,910,719 | (6.7%) | 149,643 | (38.1%) | 1,761,076 | (6.3%) |
| Chronic Obstructive Pulmonary Disease | 1,750,439 | (6.2%) | 61,559 | (15.7%) | 1,688,880 | (6.0%) |
| Peripheral Vascular Disease | 1,670,506 | (5.9%) | 87,856 | (22.3%) | 1,582,650 | (5.7%) |
| Cerebrovascular Disease | 812,635 | (2.9%) | 50,520 | (12.8%) | 762,115 | (2.7%) |
| History of Myocardial Infarction | 599,614 | (2.1%) | 20,563 | (5.2%) | 575,051 | (2.1%) |
| Dementia | 634,339 | (2.2%) | 42,387 | (10.8%) | 591,952 | (2.1%) |
| Hemiplegia or Paraplegia | 574,671 | (2.0%) | 36,507 | (9.3%) | 538,164 | (1.9%) |
| Liver disease | 546,908 | (2.0%) | 37,722 | (9.6%) | 509,186 | (1.8%) |
| Mild | 418,008 | (1.5%) | 20,950 | (5.3%) | 397,058 | (1.4%) |
| Moderate to severe | 128,900 | (0.5%) | 16,772 | (4.3%) | 112,128 | (0.4%) |
| Rheumatoid Disease | 292,112 | (1.0%) | 6,247 | (1.6%) | 285,865 | (1.0%) |
| Peptic Ulcer Disease | 230,826 | (0.8%) | 25,088 | (6.4%) | 205,738 | (0.7%) |
| AIDS/HIV | 9,179 | (<0.1%) | 424 | (0.1%) | 8,755 | (<0.1%) |
| High-Risk Surgery | 9,546,235 | (33.7%) | 297,756 | (75.7%) | 9,248,479 | (33.1%) |
| Abdominal Surgery | 6,596,343 | (23.3%) | 165,936 | (42.2%) | 6,430,407 | (23.0%) |
| Thoracic Surgery^a^ | 1,530,479 | (5.4%) | 76,981 | (19.6%) | 1,453,498 | (5.2%) |
| Cardiac Surgery | 1,101,545 | (3.9%) | 29,954 | (7.6%) | 1,071,591 | (3.8%) |
| Intracranial Surgery | 304,022 | (1.1%) | 24,115 | (6.1%) | 279,907 | (1.0%) |
| Transplantation Surgery | 13,846 | (<0.1%) | 770 | (0.2%) | 13,076 | (0.5%) |
| Postoperative Organ Injury | 1,245,898 | (4.4%) | 293,122 | (74.6%) | 1,189,122 | (4.3%) |
| Delirium | 423,649 | (1.5%) | 45,876 | (11.7%) | 377,773 | (1.4%) |
| Stroke | 167,695 | (0.6%) | 22,041 | (5.6%) | 145,654 | (0.5%) |
| Acute Myocardial Infarction | 175,556 | (0.6%) | 27,359 | (7.0%) | 148,197 | (0.5%) |
| Acute Respiratory Distress Syndrome | 29,312 | (0.1%) | 13,092 | (3.3%) | 16,220 | (<0.1%) |
| Pulmonary Embolism | 71,633 | (0.3%) | 14,359 | (3.7%) | 57,274 | (0.2%) |
| Liver Injury | 37,961 | (0.1%) | 26,089 | (6.6%) | 11,872 | (<0.1%) |
| Acute Kidney Injury | 576,438 | (20.3%) | 144,306 | (36.7%) | 432,132 | (1.5%) |

^a^without cardiac surgery;

AIDS: acquired immune deficiency syndrome; HIV: Human immunodeficiency virus, IQR: interquartile range.

## Table S2: Perioperative organ injury, in-hospital mortality and HLOS of 28,350,953 surgical patients categorized by type of surgery.

|  | Nervous System Surgery  (n=1,634,472) | Endocrine Surgery  (n=340,603) | ENT and Maxillofacial Surgery  (n=3,524,102) | Bronchial and Lung Surgery  (n=402,578) | Cardiac Surgery  (n=1,101,545) | Vascular Surgery  (n=1,633,462) | Gastrointestinal Surgery  (n=5,896,118) | Urogenital Surgery  (n=3,712,888) | Obsetric Surgery  (n=2,470,485) | Musculoskeletal Surgery  (n=8,092,551) | Breast and Skin Surgery  (n=3,473,678) |
| --- | --- | --- | --- | --- | --- | --- | --- | --- | --- | --- | --- |
| Perioperative Organ Injury | | | | | | | | | | | |
| Any Organ Injury | 80,629  (4.9%) | 6,000  (1.8%) | 138,472  (3.9%) | 59,511  (14.8%) | 207,308  (18.8%) | 200,876  (12.3%) | 398,481  (6.8%) | 134,756  (5.0%) | 993  (<0.1%) | 310,056  (3.8%) | 188,480  (5.4%) |
| Delirium | 35,405  (2.2%) | 2,262  (0.7%) | 46,122  (1.3%) | 22,415  (5.6%) | 64,868  (5.9%) | 54,580  (3.3%) | 114,058  (1.9%) | 38,040  (1.0%) | 121  (<0.1%) | 157,647  (1.9%) | 75,055  (2.2%) |
| Stroke | 24,149  (1.5%) | 591  (0.2%) | 25,564  (0.7%) | 4,301  (1.1%) | 30,912  (2.8%) | 38,896  (2.4%) | 51,329  (0.9%) | 7,935  (0.2%) | 119  (<0.1%) | 20,581  (0.25%) | 15,922  (0.5%) |
| Acute Myocardial Infarction | 4,393  (0.3%) | 909  (0.3%) | 20,408  (0.6%) | 9,518  (2.4%) | 82,693  (7.5%) | 27,906  (1.7%) | 32,971  (0.6%) | 8,108  (0.2%) | 46  (<0.1%) | 26,859  (0.3%) | 20,072  (0.6%) |
| Acute Respiratory Distress Syndrome | 2,162  (0.1%) | 288  (0.8%) | 16,379  (0.5%) | 4,056  (1.0%) | 3,218  (0.3%) | 4,745  (0.3%) | 12,709  (0.2%) | 2,269  (0.1 %) | 95  (<0.1%) | 4,325  (0.1%) | 5,744  (0.2%) |
| Pulmonary Embolism | 6,208  (0.4%) | 533  (0.2%) | 9,013  (0.3%) | 5,416  (1.3%) | 3,874  (0.4%) | 11,359  (0.7%) | 30,007  (0.5%) | 8,445  (0.2%) | 115  (<0.1%) | 16,985  (0.2%) | 9,920  (0.3%) |
| Acute Liver Injury | 1,523  (<0.1%) | 249  (<0.1%) | 8,171  (0.2%) | 3,943  (1.0%) | 5,321  (0.5%) | 8,812  (0.5%) | 22,164  (0.4%) | 2,925  (<0.1%) | 76  (<0.1%) | 6,318  (<0.1%) | 7,621  (0.2%) |
| Acute Kidney Injury | 20,378  (1.2%) | 2,689  (0.8%) | 71,516  (2.0%) | 30,442  (7.6%) | 67,629  (6.1%) | 101,900  (6.2 %) | 224,471  (3.8%) | 88,057  (2.4%) | 571  (<0.1%) | 128,611  (1.6%) | 98,438  (2.8%) |
| Outcome | | | | | | | | | | | |
| In-Hospital Mortality | 32,387  (2.0%) | 1,506  (0.4%) | 52,465  (1.5%) | 29,443  (7.3%) | 29,954  2.7%) | 70,178  (4.3%) | 167,885  (2.8%) | 30,590  (0.8%) | 110  (<0.1%) | 93,365  (1.2%) | 69,968  (2.0%) |
| Hospital Length of Stay | 4 [7-12] | 3 [3-5] | 3 [2-5] | 12 [7-21] | 9 [3-14] | 7 [3-16] | 5 [2-10] | 4 [2-7] | 4 [3-5] | 6 [3-11] | 5 [2-12] |

## Table S3: Types and subtypes of perioperative organ injury in 28,350,953 hospitalized surgical patients.

| Delirium | 423,649 | (1.5%)^b^ |
| --- | --- | --- |
| Delirium without dementia | 95,086 | (22.4%)^c^ |
| Delirium in the case of dementia | 83,057 | (19.6%)^c^ |
| Other forms of delirium^a^ | 219,028 | (51.7%)^c^ |
| Delirium not specified | 26,478 | (6.2%)^c^ |
| Stroke | 167,695 | (0.6%)^b^ |
| By thrombosis of precerebral arteries | 9,162 | (5.5%)^d^ |
| By embolism of precerebral arteries | 6,432 | (3.8%)^d^ |
| By not specified event in precerebral arteries | 8,391 | (5.0%)^d^ |
| By thrombosis of cerebral arteries | 21,721 | (13.0%)^d^ |
| By embolism of cerebral arteries | 47,561 | (28.4%)^d^ |
| By not specified event in cerebral arteries | 33,322 | (19.9%)^d^ |
| Other strokes | 20,953 | (12.5%)^d^ |
| Stroke, not specified | 12,793 | (7.6%)^d^ |
| Stroke, neither bleeding nor infarction | 7,360 | (4.4%)^d^ |
| Acute Myocardial infarction | 175,556 | (0.6%)^b^ |
| Transmural anterior myocardial infarction | 18,426 | (10.5%)^e^ |
| Transmural posterior myocardial infarction | 15,074 | (8.6%)^e^ |
| Transmural myocardial infarction of other location | 3,519 | (2.0%)^e^ |
| Transmural myocardial infarction, location not specified | 1,967 | (1.1%)^e^ |
| Subendocardial myocardial infarction | 128,692 | (73.3%)^e^ |
| myocardial infarction, not specified | 6,997 | (4.0%)^e^ |
| Acute Myocardial reinfarction | 881 | (0.5%)^e^ |
| Acute respiratory distress syndrome | 29,312 | (0.1%)^b^ |
| Mild ARDS | 1,823 | (6.2%)^f^ |
| Moderate ARDS | 5,006 | (17.1%)^f^ |
| Severe ARDS | 13,483 | (46.0%)^f^ |
| Severity not specified | 9,000 | (30.7%)^f^ |
| Pulmonary embolism | 71,633 | (0.3%)^b^ |
| With acute cor pulmonale | 17,539 | (24.5%)^g^ |
| Without acute cor pulmonale | 54,049 | (75.5%)^g^ |
| Liver Injury | 37,961 | (0.1%)^b^ |
| Toxic liver disease with acute decompensation | 570 | (1.5%)^h^ |
| Acute and subacute liver injury | 37,391 | (98.5%)^h^ |
| Acute Kidney Injury | 576,438 | (2.0%)^b^ |
| Stage 1 AKI | 159,766 | (27.7%)^k^ |
| Stage 2 AKI | 88,702 | (15.4%)^k^ |
| Stage 3 AKI | 157,012 | (27.2%)^k^ |
| Not specified | 170,958 | (29.7%)^k^ |

^a^of different etiology, including postoperative delirium; ^b^percentage of all 28,350,953 surgical patients; ^c^percentage of all 423,649 patients with perioperative delirium; ^d^percentage of all 167,695 patients with perioperative stroke; ^e^percentage of all 175,556 patients with perioperative acute myocardial infarction; ^f^percentage of all 29,312 patients with acute respiratory distress syndrome(ARDS); ^g^percentage of all 71,633 patients with pulmonary embolism; ^h^percentage of all 68,213 patients with perioperative liver injury; ^k^percentage of all 576,438 patients with perioperative acute kidney injury (AKI)

## Table S4: Four models in 28,350,953 patients describing the association of any perioperative organ injury with in-hospital mortality and morbidity.

|  | Mortality | | | | | | Morbidity | | | | | | |
| --- | --- | --- | --- | --- | --- | --- | --- | --- | --- | --- | --- | --- | --- |
|  | In-Hospital Death^a^ | | | Survival^b^ | | | Hospital Length of Stay^c^ | | | Discharge^d^ | | | |
|  | OR | (95%CI) | *P* | HR^e^ | (95%CI) | *P* | beta | (95%CI) | *P* | | HR^f^ | (95%CI) | *P* |
| Age | 1.04 | (1.04 – 1.04) | <0.001 | 1.01 | (1.01 – 1.01) | <0.001 | 0.04 | (0.04 – 0.04) | <0.001 | | 0.99 | (0.99 – 0.99) | <0,001 |
| Female sex | 0.94 | (0.93 – 0.95) | <0.001 | 0.95 | (0.94 – 0.96) | <0.001 | 0.32 | (0.32 – 0.33) | <0.001 | | 0.95 | (0.95 – 0.95) | <0,001 |
| Emergency admission | 2.09 | (2.07 – 2.10) | <0.001 | 1.65 | (1.63 – 1.66) | <0.001 | 2.02 | (2.02 – 2.03) | <0.001 | | 0.74 | (0.74 – 0.74) | <0,001 |
| Charlson Comorbidity Score Items |  |  |  |  |  |  |  |  |  | |  |  |  |
| Myocardial Infarction | 0.93 | (0.91 – 0.94) | <0.001 | 1.02 | (1.00 – 1.03) | <0.001 | -0.49 | (-0.52 – -0.46) | <0.001 | | 1.03 | (1.02 – 1.03) | <0,001 |
| Chronic Heart Failure | 2.47 | (2.45 – 2.49) | <0.001 | 1.68 | (1.67 – 1.69) | <0.001 | 3.94 | (3.92 – 3.97) | <0.001 | | 0.71 | (0.71 – 0.72) | <0,001 |
| Peripheral Vascular Disease | 1.80 | (1.78 – 1.81) | <0.001 | 1.31 | (1.30 – 1.32) | <0.001 | 3.94 | (3.92 – 3.97) | <0.001 | | 0.67 | (0.67 – 0.67) | <0,001 |
| Cerebrovascular Vascular Diesasee | 1.24 | (1.22 – 1.25) | <0.001 | 1.17 | (1.15 – 1.18) | <0.001 | 0.16 | (0.12 – 0.2) | <0.001 | | 0.94 | (0.94 – 0.95) | <0,001 |
| Dementia | 1.47 | (1.45 – 1.48) | <0.001 | 1.69 | (1.68 – 1.71) | <0.001 | 0.45 | (0.42 – 0.48) | <0.001 | | 0.88 | (0.88 – 0.88) | <0,001 |
| Chronic Pulmonary Disease | 1.34 | (1.33 – 1.35) | <0.001 | 1.11 | (1.10 – 1.12) | <0.001 | 1.54 | (1.52 – 1.56) | <0.001 | | 0.85 | (0.85 – 0.85) | <0,001 |
| Rheumatic Disease | 1.06 | (1.03 – 1.09) | <0.001 | 0.87 | (0.84 – 0.89) | <0.001 | 2.71 | (2.66 – 2.75) | <0.001 | | 0.73 | (0.73 – 0.73) | <0,001 |
| Peptic Ulcer Disease | 1.81 | (1.78 – 1.84) | <0.001 | 1.37 | (1.35 – 1.38) | <0.001 | 3.85 | (3.78 – 3.93) | <0.001 | | 0.70 | (0.70 – 0.70) | <0,001 |
| Mild Liver Disease | 1.84 | (1.81 – 1.87) | <0.001 | 1.31 | (1.30 – 1.33) | <0.001 | 2.45 | (2.40 – 2.5) | <0.001 | | 0.75 | (0.75 – 0.75) | <0,001 |
| Moderate to Severe Liver Disease | 4.71 | (4.62 – 4.81) | <0.001 | 2.55 | (2.51 – 2.59) | <0.001 | 1.83 | (1.74 – 1.93) | <0.001 | | 0.75 | (0.74 – 0.75) | <0,001 |
| Diabetes without Complications | 1.05 | (1.04 – 1.06) | <0.001 | 1.00 | (0.99 – 1.01) | <0.001 | 0.93 | (0.91 – 0.94) | <0.001 | | 0.88 | (0.88 – 0.88) | <0,001 |
| Diabetes with Complications | 0.94 | (0.93 – 0.96) | <0.001 | 0.82 | (0.81 – 0.83) | <0.001 | 2.55 | (2.52 – 2.59) | <0.001 | | 0.80 | (0.79 – 0.80) | <0,001 |
| Paraplegia or Hemiplegia | 1.05 | (1.04 – 1.07) | <0.001 | 0.78 | (0.77 – 0.79) | <0.001 | 6.97 | (6.90 – 7.03) | <0.001 | | 0.59 | (0.58 – 0.59) | <0,001 |
| Renal Disease | 1.20 | (1.19 – 1.21) | <0.001 | 1.20 | (1.19 – 1.21) | <0.001 | 1.32 | (1.30 – 1.34) | <0.001 | | 0.84 | (0.84 – 0.84) | <0,001 |
| Cancer | 1.65 | (1.64 – 1.67) | <0.001 | 1.35 | (1.34 – 1.36) | <0.001 | 1.68 | (1.66 – 1.69) | <0.001 | | 0.78 | (0.77 – 0.78) | <0,001 |
| Metastatic Cancer | 4.28 | (4.23 – 4.32) | <0.001 | 2.44 | (2.42 – 2.47) | <0.001 | 4.69 | (4.66 – 4.71) | <0.001 | | 0.56 | (0.56 – 0.56) | <0,001 |
| AIDS/HIV | 3.55 | (3.18 – 3.97) | <0.001 | 1.22 | (1.11 – 1.35) | <0.001 | 5.64 | (5.21 – 6.07) | <0.001 | | 0.62 | (0.61 – 0.64) | <0,001 |
| High-Risk Surgery |  |  |  |  |  |  |  |  |  | |  |  |  |
| Intracranial | 6.86 | (6.74 – 6.97) | <0.001 | 2.71 | (2.67 – 2.75) | <0.001 | 6.37 | (6.31 – 6.43) | <0.001 | | 0.47 | (0.47 – 0.47) | <0,001 |
| Thoracic | 1.97 | (1.95 – 1.99) | <0.001 | 1.16 | (1.15 – 1.17) | <0.001 | 3.47 | (3.44 – 3.49) | <0.001 | | 0.71 | (0.71 – 0.71) | <0,001 |
| Abdominal | 1.93 | (1.91 – 1.94) | <0.001 | 1.33 | (1.32 – 1.34) | <0.001 | 1.01 | (1.00 – 1.02) | <0.001 | | 0.93 | (0.93 – 0.93) | <0,001 |
| Cardiac Surgery | 1.02 | (1.00 – 1.03) | 0.013 | 0.96 | (0.95 – 0.97) | <0.001 | 0.06 | (0.04 – 0.09) | <0.001 | | 0.85 | (0.85 – 0.85) | <0,001 |
| Transplantation Surgery | 1.45 | (1.34 – 1.58) | <0.001 | 0.35 | (0.33 – 0.38) | <0.001 | 27.20 | (26.41 – 27.98) | <0.001 | | 0.31 | (0.31 – 0.32) | <0,001 |
| Perioperative Organ Injury | 9.28 | (9.22 – 9.35) | <0.001 | 3.51 | (3.49 – 3.54) | <0.001 | 11.15 | (11.11 – 11.19) | <0.001 | | 0.45 | (0.45 – 0.45) | <0,001 |
| Constant^g^ | 0.00 | (0.00 – 0.00) | <0.001 |  |  |  | 2.02 | (2.01 – 2.03) | <0.001 | |  |  |  |

On the left, a binary logistic regression model estimating the OR and a corresponding Cox proportional hazard regression model estimating the HR for all-cause in-hospital death; on the right, a robust regression model estimating the coefficients for hospital length of stay and a corresponding Cox proportional hazard model calculating the HR for hospital discharge.

^a^10-fold cross-validation showed a mean AUROC of 0.94 with SD <0.01; ^b^a total of 27,957,796 cases were censored due to hospital discharge; ^c^10-fold cross-validation showed a mean RMSE of 8.9 days with a SD of 0.03; ^d^393,157 cases were censored due to in-hospital death; ^e^HR for in-hospital death; ^f^HR for discharge from hospital; ^g^not applicable for Cox proportional hazard regression models.

AIDS/HIV: Acquired Immune Deficiency Syndrome, Humane Immunodeficiency Virus; HR: hazard ratio; OR: odds ratio.

## Table S5: Comparison of perioperative outcome between patients with no organ injury and different numbers of organ injuries.

| Number of Perioperative  Organ Injuries | Hospital Length of Stay  (days, median [quantiles]) | | *P* | In-hospital Mortality  %, (N) | *P* |
| --- | --- | --- | --- | --- | --- |
| No Organ Injury | 4 | [2–8] | <0.001^#^ | 0.7%  (181,573/27,079,724) | <0.001^§^ |
| 1 Organ Injury | 16 | [10–27] |  | 13.9%  (144,713/1,042,713) |  |
| 2 Organ Injuries | 24 | [14–39] |  | 31.0%  (53,974/173,959) |  |
| ≥3 Organ Injuries | 29 | [17–48] |  | 43.7%  (12,897/29,491) |  |

^#^median test, χ^2^(3)=1.1*10^6^; ^§^chi-squared test, χ^2^(3) = 2.8*10^6^.

## Table S6: Four models in 28,350,953 patients describing the association of multiple perioperative organ injuries with in-hospital mortality and morbidity.

|  | Mortality | | | | | | | Morbidity | | | | | | |
| --- | --- | --- | --- | --- | --- | --- | --- | --- | --- | --- | --- | --- | --- | --- |
|  | In-Hospital Death^a^ | | | | Survival^b^ | | | Hospital Length of Stay^c^ | | | | Discharge^d^ | | |
|  | OR | (95%CI) | *P* | HR^e^ | | (95%CI) | *P* | beta | (95%CI) | *P* | HR^f^ | | (95%CI) | *P* |
| Age | 1.04 | (1.04 – 1.04) | <0.001 | 1.01 | | (1.01 – 1.01) | <0.001 | 0.04 | (0.04 – 0.04) | <0.001 | 0.99 | | (0.99 – 0.99) | <0.001 |
| Sex | 0.96 | (0.95 – 0.96) | <0.001 | 0.96 | | (0.96 – 0.97) | <0.001 | 0.33 | (0.33 – 0.34) | <0.001 | 0.95 | | (0.95 – 0.95) | <0.001 |
| Emergency admission | 2.11 | (2.09 – 2.12) | <0.001 | 1.65 | | (1.63 – 1.66) | <0.001 | 2.03 | (2.02 – 2.04) | <0.001 | 0.74 | | (0.74 – 0.74) | <0.001 |
| Charlson Comorbidity Score Items |  |  |  |  | |  |  |  |  |  |  | |  |  |
| Myocardial Infarction | 0.93 | (0.92 – 0.95) | <0.001 | 1.02 | | (1.00 – 1.03) | 0.021 | -0.47 | (-0.5 – -0.44) | <0.001 | 1.03 | | (1.02 – 1.03) | <0.001 |
| Chronic Heart Failure | 2.38 | (2.36 – 2.4) | <0.001 | 1.65 | | (1.64 – 1.66) | <0.001 | 3.80 | (3.77 – 3.82) | <0.001 | 0.72 | | (0.72 – 0.72) | <0.001 |
| Peripheral Vascular Disease | 1.79 | (1.78 – 1.81) | <0.001 | 1.30 | | (1.29 – 1.31) | <0.001 | 3.91 | (3.88 – 3.93) | <0.001 | 0.67 | | (0.67 – 0.67) | <0.001 |
| Cerebrovascular Diesasee | 1.24 | (1.22 – 1.25) | <0.001 | 1.16 | | (1.15 – 1.17) | <0.001 | 0.15 | (0.11 – 0.19) | <0.001 | 0.94 | | (0.94 – 0.95) | <0.001 |
| Dementia | 1.50 | (1.49 – 1.52) | <0.001 | 1.70 | | (1.69 – 1.72) | <0.001 | 0.51 | (0.48 – 0.54) | <0.001 | 0.88 | | (0.87 – 0.88) | <0.001 |
| Chronic Pulmonary Disease | 1.34 | (1.33 – 1.36) | <0.001 | 1.10 | | (1.09 – 1.11) | <0.001 | 1.53 | (1.51 – 1.55) | <0.001 | 0.85 | | (0.85 – 0.85) | <0.001 |
| Rheumatic Disease | 1.06 | (1.03 – 1.09) | <0.001 | 0.86 | | (0.84 – 0.89) | <0.001 | 2.71 | (2.66 – 2.75) | <0.001 | 0.73 | | (0.73 – 0.73) | <0.001 |
| Peptic Ulcer Disease | 1.77 | (1.74 – 1.8) | <0.001 | 1.35 | | (1.33 – 1.37) | <0.001 | 3.75 | (3.68 – 3.82) | <0.001 | 0.70 | | (0.70 – 0.71) | <0.001 |
| Mild Liver Disease | 1.82 | (1.79 – 1.85) | <0.001 | 1.30 | | (1.28 – 1.32) | <0.001 | 2.39 | (2.34 – 2.43) | <0.001 | 0.75 | | (0.75 – 0.75) | <0.001 |
| Moderate to Severe Liver Disease | 4.60 | (4.51 – 4.69) | <0.001 | 2.50 | | (2.46 – 2.54) | <0.001 | 1.67 | (1.57 – 1.76) | <0.001 | 0.75 | | (0.75 – 0.76) | <0.001 |
| Diabetes without complications | 1.04 | (1.03 – 1.05) | <0.001 | 0.99 | | (0.99 – 1.00) | 0.157 | 0.92 | (0.91 – 0.93) | <0.001 | 0.88 | | (0.88 – 0.88) | <0.001 |
| Diabetes with complications | 0.94 | (0.93 – 0.95) | <0.001 | 0.82 | | (0.81 – 0.83) | <0.001 | 2.55 | (2.52 – 2.59) | <0.001 | 0.80 | | (0.79 – 0.80) | <0.001 |
| Paraplegia or Hemiplegia | 1.00 | (0.99 – 1.02) | 0.712 | 0.77 | | (0.76 – 0.78) | <0.001 | 6.87 | (6.81 – 6.94) | <0.001 | 0.59 | | (0.58 – 0.59) | <0.001 |
| Renal Disease | 1.22 | (1.21 – 1.23) | <0.001 | 1.21 | | (1.20 – 1.21) | <0.001 | 1.33 | (1.31 – 1.35) | <0.001 | 0.84 | | (0.84 – 0.84) | <0.001 |
| Cancer | 1.68 | (1.66 – 1.7) | <0.001 | 1.35 | | (1.34 – 1.37) | <0.001 | 1.68 | (1.67 – 1.7) | <0.001 | 0.78 | | (0.77 – 0.78) | <0.001 |
| Metastatic Cancer | 4.39 | (4.34 – 4.44) | <0.001 | 2.47 | | (2.44 – 2.49) | <0.001 | 4.71 | (4.68 – 4.73) | <0.001 | 0.56 | | (0.56 – 0.56) | <0.001 |
| AIDS/HIV | 3.53 | (3.15 – 3.95) | <0.001 | 1.22 | | (1.11 – 1.34) | <0.001 | 5.55 | (5.12 – 5.97) | <0.001 | 0.63 | | (0.61 – 0.64) | <0.001 |
| Type of surgery |  |  |  |  | |  |  |  |  |  |  | |  |  |
| Intracranial | 7.15 | (7.03 – 7.27) | <0.001 | 2.73 | | (2.70 – 2.77) | <0.001 | 6.40 | (6.34 – 6.47) | <0.001 | 0.47 | | (0.47 – 0.47) | <0.001 |
| Thoracic | 1.96 | (1.94 – 1.98) | <0.001 | 1.15 | | (1.14 – 1.16) | <0.001 | 3.45 | (3.42 – 3.48) | <0.001 | 0.71 | | (0.71 – 0.71) | <0.001 |
| Abdominal | 1.90 | (1.89 – 1.92) | <0.001 | 1.32 | | (1.31 – 1.33) | <0.001 | 0.99 | (0.98 – 1.00) | <0.001 | 0.93 | | (0.93 – 0.93) | <0.001 |
| Cardiac Surgery | 0.94 | (0.93 – 0.95) | <0.001 | 0.94 | | (0.92 – 0.95) | <0.001 | 0.03 | (0.00 – 0.05) | <0.001 | 0.85 | | (0.85 – 0.85) | <0.001 |
| Transplantation Surgery | 1.23 | (1.13 – 1.35) | <0.001 | 0.34 | | (0.32 – 0.36) | <0.001 | 26.84 | (26.06 – 27.62) | <0.001 | 0.31 | | (0.31 – 0.32) | <0.001 |
| Number of perioperative organ injuries |  |  |  |  | |  |  |  |  |  |  | |  |  |
| 1 POIs | 7.36 | (7.31 – 7.42) | <0.001* | 3.23 | | (3.20 – 3.25) | <0.001* | 9.81 | (9.77 – 9.85) | <0.001* | 0.48 | | (0.48 – 0.48) | <0.001* |
| 2 POIs | 19.73 | (19.49 – 19.98) | <0.001* | 4.55 | | (4.50 – 4.60) | <0.001* | 17.45 | (17.32 – 17.57) | <0.001* | 0.32 | | (0.32 – 0.32) | <0.001* |
| ≥3 POIs | 41.67 | (40.58 – 42.79) | <0.001* | 5.30 | | (5.20 – 5.40) | <0.001* | 23.69 | (23.32 – 24.06) | <0.001* | 0.23 | | (0.22 – 0.23) | <0.001* |
| Constant^g^ | 0.00 | (0.00 – 0.00) | <0.001 |  | |  |  | 1.99 | (1.98 – 2.01) | <0.001 |  | |  |  |

On the left, a binary logistic regression model estimating the OR and a corresponding Cox proportional hazard regression model estimating the HR for all-cause in-hospital death; on the right, a robust regression model estimating the coefficients for hospital length of stay and a corresponding Cox proportional hazard model calculating the HR for hospital discharge.

^a^10-fold cross-validation showed a mean AUROC of 0.94 with SD <0.01; ^b^a total of 27,957,796 cases were censored due to hospital discharge; ^c^10-fold cross-validation showed a mean RMSE of 8.9 days with a SD of 0.04; ^d^393,157 cases were censored due to in-hospital death; ^e^HR for in-hospital death; ^f^HR for discharge from hospital; ^g^not applicable for Cox proportional hazard regression models.

AIDS/HIV: Acquired Immune Deficiency Syndrome, Humane Immunodeficiency Virus; HR: hazard ratio; OR: odds ratio.

* refers to the comparison of 1, 2, or 3 and more organ injuries with no organ injury respectively

## Table S7: Four models in 28,350,953 patients describing the association of individual perioperative organ injuries with in-hospital mortality and morbidity.

|  | Mortality | | | | | | Morbidity | | | | | |
| --- | --- | --- | --- | --- | --- | --- | --- | --- | --- | --- | --- | --- |
|  | In-Hospital Death^a^ | | | Survival^b^ | | | Hospital Length of Stay^c^ | | | Discharge^d^ | | |
|  | OR | (95%CI) | *P* | HR^e^ | (95%CI) | *P* | beta | (95%CI) | *P* | HR^f^ | (95%CI) | *P* |
| Age | 1.04 | (1.04 – 1.04) | <0.001 | 1.01 | (1.01 – 1.01) | <0.001 | 0.04 | (0.04 – 0.04) | <0.001 | 0.99 | (0.99 – 0.99) | <0.001 |
| Sex | 0.93 | (0.93 – 0.94) | <0.001 | 0.94 | (0.93 – 0.94) | <0.001 | 0.34 | (0.33 – 0.34) | <0.001 | 0.95 | (0.95 – 0.95) | <0.001 |
| Emergency hospital admission | 2.20 | (2.19 – 2.22) | <0.001 | 1.67 | (1.66 – 1.68) | <0.001 | 2.04 | (2.03 – 2.05) | <0.001 | 0.74 | (0.74 – 0.74) | <0.001 |
| Charlson Comorbidity Score Items |  |  |  |  |  |  |  |  |  |  |  |  |
| Myocardial Infarction | 0.94 | (0.92 – 0.96) | <0.001 | 1.03 | (1.02 – 1.05) | <0.001 | -0.46 | (-0.49 – -0.43) | <0.001 | 1.03 | (1.02 – 1.03) | <0.001 |
| Chronic Heart Failure | 2.40 | (2.38 – 2.43) | <0.001 | 1.64 | (1.63 – 1.66) | <0.001 | 3.81 | (3.79 – 3.84) | <0.001 | 0.72 | (0.72 – 0.72) | <0.001 |
| Peripheral Vascular Disease | 1.84 | (1.83 – 1.86) | <0.001 | 1.31 | (1.30 – 1.32) | <0.001 | 3.92 | (3.90 – 3.95) | <0.001 | 0.67 | (0.67 – 0.67) | <0.001 |
| Cerebrovascular Vascular Diesasee | 1.35 | (1.33 – 1.36) | <0.001 | 1.22 | (1.21 – 1.24) | <0.001 | 0.21 | (0.17 – 0.25) | <0.001 | 0.94 | (0.93 – 0.94) | <0.001 |
| Dementia | 1.84 | (1.81 – 1.86) | <0.001 | 2.04 | (2.01 – 2.06) | <0.001 | 0.41 | (0.38 – 0.44) | <0.001 | 0.87 | (0.87 – 0.87) | <0.001 |
| Chronic Pulmonary Disease | 1.36 | (1.35 – 1.38) | <0.001 | 1.13 | (1.12 – 1.14) | <0.001 | 1.50 | (1.48 – 1.52) | <0.001 | 0.85 | (0.85 – 0.85) | <0.001 |
| Rheumatic Disease | 1.04 | (1.01 – 1.07) | 0.003 | 0.86 | (0.84 – 0.88) | <0.001 | 2.70 | (2.66 – 2.74) | <0.001 | 0.73 | (0.73 – 0.73) | <0.001 |
| Peptic Ulcer Disease | 1.78 | (1.75 – 1.81) | <0.001 | 1.33 | (1.31 – 1.34) | <0.001 | 3.74 | (3.67 – 3.81) | <0.001 | 0.70 | (0.70 – 0.71) | <0.001 |
| Mild Liver Disease | 1.63 | (1.60 – 1.66) | <0.001 | 1.20 | (1.19 – 1.22) | <0.001 | 2.37 | (2.32 – 2.41) | <0.001 | 0.75 | (0.75 – 0.76) | <0.001 |
| Moderate to Severe Liver Disease | 3.88 | (3.80 – 3.97) | <0.001 | 2.06 | (2.02 – 2.09) | <0.001 | 1.69 | (1.59 – 1.78) | <0.001 | 0.77 | (0.76 – 0.77) | <0.001 |
| Diabetes without complications | 1.03 | (1.02 – 1.04) | <0.001 | 0.99 | (0.99 – 1.00) | 0.131 | 0.93 | (0.92 – 0.95) | <0.001 | 0.88 | (0.88 – 0.88) | <0.001 |
| Diabetes with complications | 0.93 | (0.91 – 0.94) | <0.001 | 0.81 | (0.80 – 0.82) | <0.001 | 2.55 | (2.52 – 2.59) | <0.001 | 0.80 | (0.79 – 0.8 ) | <0.001 |
| Paraplegia or Hemiplegia | 1.23 | (1.21 – 1.25) | <0.001 | 0.87 | (0.86 – 0.88) | <0.001 | 7.30 | (7.23 – 7.36) | <0.001 | 0.57 | (0.56 – 0.57) | <0.001 |
| Renal Disease | 1.21 | (1.20 – 1.22) | <0.001 | 1.22 | (1.21 – 1.23) | <0.001 | 1.29 | (1.27 – 1.31) | <0.001 | 0.84 | (0.84 – 0.85) | <0.001 |
| Cancer | 1.65 | (1.63 – 1.67) | <0.001 | 1.35 | (1.34 – 1.36) | <0.001 | 1.66 | (1.65 – 1.68) | <0.001 | 0.78 | (0.78 – 0.78) | <0.001 |
| Metastatic Cancer | 4.40 | (4.36 – 4.45) | <0.001 | 2.45 | (2.43 – 2.48) | <0.001 | 4.68 | (4.66 – 4.71) | <0.001 | 0.56 | (0.56 – 0.56) | <0.001 |
| AIDS/HIV | 3.35 | (2.98 – 3.78) | <0.001 | 1.16 | (1.05 – 1.27) | 0.018 | 5.43 | (5.00 – 5.85) | <0.001 | 0.63 | (0.62 – 0.65) | <0.001 |
| High-Risk Surgery |  |  |  |  |  |  |  |  |  |  |  |  |
| Intracranial | 8.37 | (8.23 – 8.51) | <0.001 | 2.95 | (2.91 – 3.00) | <0.001 | 6.42 | (6.36 – 6.48) | <0.001 | 0.47 | (0.47 – 0.47) | <0.001 |
| Thoracic | 1.97 | (1.95 – 1.99) | <0.001 | 1.14 | (1.13 – 1.15) | <0.001 | 3.46 | (3.43 – 3.49) | <0.001 | 0.71 | (0.71 – 0.71) | <0.001 |
| Abdominal | 1.85 | (1.83 – 1.86) | <0.001 | 1.31 | (1.30 – 1.32) | <0.001 | 0.99 | (0.98 – 1.00) | <0.001 | 0.93 | (0.93 – 0.93) | <0.001 |
| Cardiac Surgery | 1.00 | (0.98 – 1.01) | 0.974 | 0.96 | (0.95 – 0.98) | <0.001 | 0.35 | (0.32 – 0.38) | <0.001 | 0.84 | (0.84 – 0.84) | <0.001 |
| Transplantation Surgery | 0.95 | (0.86 – 1.05) | 0.295 | 0.30 | (0.28 – 0.33) | <0.001 | 26.84 | (26.06 – 27.61) | <0.001 | 0.32 | (0.31 – 0.32) | <0.001 |
| Type of perioperative organ injuries |  |  |  |  |  |  |  |  |  |  |  |  |
| Delirium | 1.40 | (1.39 – 1.42) | <0.001* | 0.81 | (0.8 – 0.82) | <0.001* | 10.59 | (10.53 – 10.66) | <0.001* | 0.54 | (0.54 – 0.54) | <0.001* |
| Stroke | 2.99 | (2.93 – 3.05) | <0.001* | 1.56 | (1.54 – 1.58) | <0.001* | 6.47 | (6.35 – 6.59) | <0.001* | 0.67 | (0.67 – 0.67) | <0.001* |
| Acute Myocardial Infarction | 3.28 | (3.23 – 3.34) | <0.001* | 1.82 | (1.80 – 1.85) | <0.001* | 4.28 | (4.2 – 4.37) | <0.001* | 0.65 | (0.65 – 0.66) | <0.001* |
| Acute Respiratory Distress Syndrome | 10.60 | (10.28 – 10.94) | <0.001* | 1.75 | (1.72 – 1.78) | <0.001* | 16.04 | (15.67 – 16.41) | <0.001* | 0.32 | (0.31 – 0.32) | <0.001* |
| Pulmonary Embolism | 5.27 | (5.15 – 5.39) | <0.001* | 1.88 | (1.85 – 1.92) | <0.001* | 9.01 | (8.85 – 9.17) | <0.001* | 0.48 | (0.47 – 0.48) | <0.001* |
| Liver Injury | 24.96 | (24.28 – 25.65) | <0.001* | 3.48 | (3.43 – 3.53) | <0.001* | 3.01 | (2.71 – 3.30) | <0.001* | 0.31 | (0.30 – 0.31) | <0.001* |
| Acute Kidney Injury | 7.91 | (7.84 – 7.98) | <0.001* | 3.15 | (3.12 – 3.17) | <0.001* | 10.24 | (10.18 – 10.30) | <0.001* | 0.46 | (0.46 – 0.46) | <0.001* |
| Constant^g^ | 0.00 | (0.00 – 0.00) | <0.001 |  |  |  | 1.98 | (1.97 – 1.99) | <0.001 |  |  |  |

On the left, a binary logistic regression model estimating the odds ratio and a corresponding Cox proportional hazard regression model estimating the hazard ratio for all-cause in-hospital death; on the right, a robust regression model estimating the coefficients for hospital length of stay and a corresponding Cox proportional hazard model calculating the hazard ratio for hospital discharge.

^a^10-fold cross-validation showed a mean AUROC of 0.94 with a SD of <0.01; ^b^A total of 27,957,796 cases were censored due to hospital discharge; ^c^10-fold cross-validation showed a mean root mean square error (RMSE) of 8.9 days with a SD of 0.02; ^d^393,157 cases were censored due to in-hospital death; ^e^HR for in-hospital death; ^f^HR for discharge from hospital; ^g^not applicable for Cox proportional hazard regression models. AIDS/HIV: Acquired Immune Deficiency Syndrome, Humane Immunodeficiency Virus; HR: hazard ratio; OR: odds ratio; SIRS: systemic inflammatory response syndrome.

* refers to the comparison of individual organ injury (such as AKI, ARDS) compared with no occurrence of that specific organ injury

## Table S8: Three sensitivity analysis models in 28,350,953 patients confirming the association of any perioperative organ injury with morbidity

|  | Morbidity | | | | | | | | |
| --- | --- | --- | --- | --- | --- | --- | --- | --- | --- |
|  | Logarithm of In-Hospital Death | | | Hospital Discharge | | | Hospital-Free Days^b^ | | |
|  | beta | (95%CI) | *P* | SHR^c^ | (95%CI) | *P* | OR | (95%CI) | *P* |
| Age | 0.01 | (0.01 – 0.01) | <0.001 | 0.99 | (0.99 – 0.99) | <0.001 | 0.98 | (0.98 – 0.98) | <0,001 |
| Female sex | 0.10 | (0.10 – 0.1)0 | <0.001 | 0.95 | (0.95 – 0.95) | <0.001 | 0.79 | (0.79 – 0.79) | <0,001 |
| Emergency admission | 0.30 | (0.30 – 0.30) | <0.001 | 0.73 | (0.73 – 0.73) | <0.001 | 0.53 | (0.53 – 0.53) | <0,001 |
| Charlson Comorbidity Score Items |  |  |  |  |  |  |  |  |  |
| Myocardial Infarction | -0.03 | (-0.03 – -0.03) | <0.001 | 1.03 | (1.02 – 1.03) | <0.001 | 1.07 | (1.06 – 1.07) | <0,001 |
| Chronic Heart Failure | 0.31 | (0.31 – 0.31) | <0.001 | 0.70 | (0.70 – 0.70) | <0.001 | 0.47 | (0.46 – 0.47) | <0,001 |
| Peripheral Vascular Disease | 0.42 | (0.42 – 0.42) | <0.001 | 0.67 | (0.67 – 0.67) | <0.001 | 0.40 | (0.40 – 0.40) | <0,001 |
| Cerebrovascular Vascular Diesasee | 0.08 | (0.08 – 0.08) | <0.001 | 0.93 | (0.93 – 0.93) | <0.001 | 0.81 | (0.81 – 0.81) | <0,001 |
| Dementia | 0.09 | (0.09 – 0.10) | <0.001 | 0.83 | (0.83 – 0.83) | <0.001 | 0.76 | (0.75 – 0.76) | <0,001 |
| Chronic Pulmonary Disease | 0.15 | (0.15 – 0.15) | <0.001 | 0.85 | (0.85 – 0.85) | <0.001 | 0.74 | (0.73 – 0.74) | <0,001 |
| Rheumatic Disease | 0.30 | (0.30 – 0.31) | <0.001 | 0.74 | (0.74 – 0.75) | <0.001 | 0.54 | (0.54 – 0.55) | <0,001 |
| Peptic Ulcer Disease | 0.36 | (0.36 – 0.36) | <0.001 | 0.67 | (0.67 – 0.67) | <0.001 | 0.42 | (0.42 – 0.43) | <0,001 |
| Mild Liver Disease | 0.26 | (0.26 – 0.26) | <0.001 | 0.74 | (0.74 – 0.75) | <0.001 | 0.58 | (0.57 – 0.58) | <0,001 |
| Moderate to Severe Liver Disease | 0.14 | (0.13 – 0.14) | <0.001 | 0.68 | (0.68 – 0.69) | <0.001 | 0.59 | (0.58 – 0.60) | <0,001 |
| Diabetes without Complications | 0.11 | (0.11 – 0.11) | <0.001 | 0.89 | (0.89 – 0.89) | <0.001 | 0.80 | (0.80 – 0.80) | <0,001 |
| Diabetes with Complications | 0.20 | (0.20 – 0.20) | <0.001 | 0.82 | (0.82 – 0.82) | <0.001 | 0.70 | (0.70 – 0.70) | <0,001 |
| Paraplegia or Hemiplegia | 0.43 | (0.43 – 0.43) | <0.001 | 0.63 | (0.62 – 0.63) | <0.001 | 0.50 | (0.50 – 0.50) | <0,001 |
| Renal Disease | 0.17 | (0.17 – 0.17) | <0.001 | 0.84 | (0.83 – 0.84) | <0.001 | 0.69 | (0.69 – 0.70) | <0,001 |
| Cancer | 0.27 | (0.27 – 0.28) | <0.001 | 0.77 | (0.77 – 0.77) | <0.001 | 0.58 | (0.58 – 0.59) | <0,001 |
| Metastatic Cancer | 0.61 | (0.61 – 0.61) | <0.001 | 0.54 | (0.53 – 0.54) | <0.001 | 0.26 | (0.26 – 0.26) | <0,001 |
| AIDS/HIV | 0.32 | (0.30 – 0.34) | <0.001 | 0.64 | (0.63 – 0.66) | <0.001 | 0.59 | (0.56 – 0.61) | <0,001 |
| High-Risk Surgery |  |  |  |  |  |  |  |  |  |
| Intracranial | 0.73 | (0.73 – 0.74) | <0.001 | 0.44 | (0.44 – 0.44) | <0.001 | 0.19 | (0.19 – 0.19) | <0,001 |
| Thoracic | 0.23 | (0.23 – 0.24) | <0.001 | 0.72 | (0.72 – 0.72) | <0.001 | 0.62 | (0.61 – 0.62) | <0,001 |
| Abdominal | 0.06 | (0.06 – 0.06) | <0.001 | 0.93 | (0.93 – 0.93) | <0.001 | 0.91 | (0.91 – 0.91) | <0,001 |
| Cardiac Surgery | 0.11 | (0.11 – 0.11) | <0.001 | 0.86 | (0.86 – 0.86) | <0.001 | 0.87 | (0.87 – 0.88) | <0,001 |
| Transplantation Surgery | 1.42 | (1.41 – 1.43) | <0.001 | 0.35 | (0.35 – 0.35) | <0.001 | 0.08 | (0.08 – 0.09) | <0,001 |
| Perioperative Organ Injury | 0.78 | (0.77 – 0.78) | <0.001 | 0.41 | (0.41 – 0.41) | <0.001 | 0.14 | (0.14 – 0.14) | <0.001 |
| Constant^g^ | 0.73 | (0.73 – 0.73) | <0.001 | --- | --- | --- | --- | --- | --- |

On the left, a linear regression model estimating the association with the logarithm of HLOS. In the middle, a competing risk model estimating the subdistribution hazard ratio for hospital discharge with death as competing risk. On the right, a proportional odds model estimating the odds ratio hospital-free of 90 days.

^a^a total of 393,157 in-hospital deaths were counted as competing risk; ^b^hospital-free days out of 90; ^c^n/a for proportional hazard and proportional odds models.

AIDS/HIV: Acquired Immune Deficiency Syndrome, Humane Immunodeficiency Virus; SHR: subdistribution hazard ratio; OR: odds ratio.

## Table S9: Three sensitivity analysis models in 28,350,953 patients confirming the association of the number of perioperative organ injuries with morbidity

|  | Morbidity | | | | | | | | |
| --- | --- | --- | --- | --- | --- | --- | --- | --- | --- |
|  | Logarithm of In-Hospital Death | | | Hospital Discharge | | | Hospital-Free Days^b^ | | |
|  | beta | (95%CI) | *P* | SHR^c^ | (95%CI) | *P* | OR | (95%CI) | *P* |
| Age | 0.01 | (0.01 – 0.01) | <0.001 | 0.99 | (0.99 – 0.99) | <0.001 | 0.98 | (0.98 – 0.98) | <0,001 |
| Female sex | 0.10 | (0.10 – 0.10) | <0.001 | 0.95 | (0.95 – 0.95) | <0.001 | 0.79 | (0.79 – 0.79) | <0,001 |
| Emergency admission | 0.30 | (0.30 – 0.30) | <0.001 | 0.73 | (0.73 – 0.73) | <0.001 | 0.53 | (0.53 – 0.53) | <0,001 |
| Charlson Comorbidity Score Items |  |  |  |  |  |  |  |  |  |
| Myocardial Infarction | -0.03 | (-0.03 – -0.03) | <0.001 | 1.03 | (1.02 – 1.03) | <0.001 | 1.07 | (1.06 – 1.07) | <0,001 |
| Chronic Heart Failure | 0.31 | (0.30 – 0.31) | <0.001 | 0.70 | (0.7 – 0.7) | <0.001 | 0.47 | (0.47 – 0.48) | <0,001 |
| Peripheral Vascular Disease | 0.42 | (0.41 – 0.42) | <0.001 | 0.67 | (0.67 – 0.67) | <0.001 | 0.40 | (0.40 – 0.40) | <0,001 |
| Cerebrovascular Vascular Diesasee | 0.08 | (0.08 – 0.08) | <0.001 | 0.93 | (0.93 – 0.93) | <0.001 | 0.81 | (0.81 – 0.81) | <0,001 |
| Dementia | 0.09 | (0.09 – 0.10) | <0.001 | 0.83 | (0.82 – 0.83) | <0.001 | 0.75 | (0.75 – 0.75) | <0,001 |
| Chronic Pulmonary Disease | 0.15 | (0.15 – 0.15) | <0.001 | 0.85 | (0.85 – 0.85) | <0.001 | 0.74 | (0.73 – 0.74) | <0,001 |
| Rheumatic Disease | 0.30 | (0.30 – 0.31) | <0.001 | 0.74 | (0.74 – 0.75) | <0.001 | 0.54 | (0.54 – 0.55) | <0,001 |
| Peptic Ulcer Disease | 0.36 | (0.35 – 0.36) | <0.001 | 0.67 | (0.67 – 0.68) | <0.001 | 0.43 | (0.42 – 0.43) | <0,001 |
| Mild Liver Disease | 0.26 | (0.26 – 0.26) | <0.001 | 0.75 | (0.74 – 0.75) | <0.001 | 0.58 | (0.58 – 0.58) | <0,001 |
| Moderate to Severe Liver Disease | 0.13 | (0.13 – 0.14) | <0.001 | 0.69 | (0.69 – 0.69) | <0.001 | 0.60 | (0.59 – 0.6) | <0,001 |
| Diabetes without Complications | 0.11 | (0.11 – 0.11) | <0.001 | 0.89 | (0.89 – 0.89) | <0.001 | 0.80 | (0.80 – 0.80) | <0,001 |
| Diabetes with Complications | 0.20 | (0.20 – 0.20) | <0.001 | 0.82 | (0.82 – 0.82) | <0.001 | 0.70 | (0.69 – 0.7) | <0,001 |
| Paraplegia or Hemiplegia | 0.43 | (0.43 – 0.43) | <0.001 | 0.63 | (0.63 – 0.63) | <0.001 | 0.51 | (0.51 – 0.51) | <0,001 |
| Renal Disease | 0.17 | (0.17 – 0.17) | <0.001 | 0.83 | (0.83 – 0.84) | <0.001 | 0.69 | (0.69 – 0.69) | <0,001 |
| Cancer | 0.27 | (0.27 – 0.28) | <0.001 | 0.77 | (0.77 – 0.77) | <0.001 | 0.58 | (0.58 – 0.59) | <0,001 |
| Metastatic Cancer | 0.61 | (0.61 – 0.61) | <0.001 | 0.54 | (0.53 – 0.54) | <0.001 | 0.26 | (0.25 – 0.26) | <0,001 |
| AIDS/HIV | 0.32 | (0.30 – 0.33) | <0.001 | 0.65 | (0.63 – 0.66) | <0.001 | 0.59 | (0.57 – 0.61) | <0,001 |
| High-Risk Surgery |  |  |  |  |  |  |  |  |  |
| Intracranial | 0.73 | (0.73 – 0.74) | <0.001 | 0.44 | (0.44 – 0.44) | <0.001 | 0.19 | (0.19 – 0.19) | <0,001 |
| Thoracic | 0.23 | (0.23 – 0.24) | <0.001 | 0.72 | (0.72 – 0.72) | <0.001 | 0.62 | (0.61 – 0.62) | <0,001 |
| Abdominal | 0.06 | (0.05 – 0.06) | <0.001 | 0.93 | (0.93 – 0.93) | <0.001 | 0.91 | (0.91 – 0.91) | <0,001 |
| Cardiac Surgery | 0.11 | (0.11 – 0.11) | <0.001 | 0.86 | (0.86 – 0.86) | <0.001 | 0.88 | (0.87 – 0.88) | <0,001 |
| Transplantation Surgery | 1.41 | (1.40 – 1.42) | <0.001 | 0.35 | (0.35 – 0.36) | <0.001 | 0.09 | (0.08 – 0.09) | <0,001 |
| Number of Perioperative Organ Injuries |  |  |  |  |  |  |  |  |  |
| 1 POIs | 0.73 | (0.73 – 0.74) | <0.001 | 0.44 | (0.44 – 0.44) | <0.001 | 0.16 | (0.16 – 0.16) | <0,001 |
| 2 POIs | 0.98 | (0.97 – 0.98) | <0.001 | 0.27 | (0.27 – 0.28) | <0.001 | 0.06 | (0.06 – 0.06) | <0,001 |
| ≥3 POIs | 1.14 | (1.13 – 1.15) | <0.001 | 0.19 | (0.19 – 0.19) | <0.001 | 0.03 | (0.03 – 0.03) | <0,001 |
| Constant^c^ | 0.73 | (0.73 – 0.73) | <0.001 | --- | --- | --- | --- | --- | --- |

On the left, a linear regression model estimating the coefficient for the logarithm of HLOS. In the middle, a competing risk model estimating the subdistribution hazard ratio for hospital discharge with death as competing risk. On the right, a proportional odds model estimating the odds ratio hospital-free of 90 days.

^a^a total of 393,157 in-hospital deaths were counted as competing risk; ^b^hospital-free days out of 90; ^c^n/a for proportional hazard and proportional odds models.

AIDS/HIV: Acquired Immune Deficiency Syndrome, Humane Immunodeficiency Virus; SHR: subdistribution hazard ratio; OR: odds ratio.

## Table S10: Three sensitivity analysis models in 28,350,953 patients confirming the association of individual perioperative organ injuries with morbidity

|  | Morbidity | | | | | | | | |
| --- | --- | --- | --- | --- | --- | --- | --- | --- | --- |
|  | Logarithm of In-Hospital Death | | | Hospital Discharge | | | Hospital-Free Days^b^ | | |
|  | beta | (95%CI) | *P* | SHR^c^ | (95%CI) | *P* | OR | (95%CI) | *P* |
| Age | 0.01 | (0.01 – 0.01) | <0.001 | 0.99 | (0.99 – 0.99) | <0.001 | 0.98 | (0.98 – 0.98) | <0,001 |
| Female sex | 0.10 | (0.1 – 0.1) | <0.001 | 0.95 | (0.95 – 0.95) | <0.001 | 0.79 | (0.79 – 0.79) | <0,001 |
| Emergency admission | 0.30 | (0.3 – 0.3) | <0.001 | 0.73 | (0.73 – 0.73) | <0.001 | 0.53 | (0.53 – 0.53) | <0,001 |
| Charlson Comorbidity Score Items |  |  |  |  |  |  |  |  |  |
| Myocardial Infarction | -0.03 | (-0.03 – -0.03) | <0.001 | 1.02 | (1.02 – 1.03) | <0.001 | 1.07 | (1.06 – 1.07) | <0,001 |
| Chronic Heart Failure | 0.31 | (0.31 – 0.31) | <0.001 | 0.70 | (0.70 – 0.70) | <0.001 | 0.48 | (0.48 – 0.48) | <0,001 |
| Peripheral Vascular Disease | 0.42 | (0.42 – 0.42) | <0.001 | 0.67 | (0.67 – 0.67) | <0.001 | 0.40 | (0.40 – 0.40) | <0,001 |
| Cerebrovascular Vascular Diesasee | 0.09 | (0.08 – 0.09) | <0.001 | 0.92 | (0.92 – 0.92) | <0.001 | 0.79 | (0.79 – 0.79) | <0,001 |
| Dementia | 0.09 | (0.09 – 0.1) | <0.001 | 0.82 | (0.81 – 0.82) | <0.001 | 0.73 | (0.72 – 0.73) | <0,001 |
| Chronic Pulmonary Disease | 0.15 | (0.15 – 0.15) | <0.001 | 0.85 | (0.85 – 0.85) | <0.001 | 0.74 | (0.74 – 0.74) | <0,001 |
| Rheumatic Disease | 0.30 | (0.3 – 0.31) | <0.001 | 0.74 | (0.74 – 0.75) | <0.001 | 0.54 | (0.54 – 0.55) | <0,001 |
| Peptic Ulcer Disease | 0.36 | (0.36 – 0.36) | <0.001 | 0.68 | (0.67 – 0.68) | <0.001 | 0.43 | (0.43 – 0.43) | <0,001 |
| Mild Liver Disease | 0.26 | (0.26 – 0.26) | <0.001 | 0.75 | (0.75 – 0.76) | <0.001 | 0.59 | (0.59 – 0.59) | <0,001 |
| Moderate to Severe Liver Disease | 0.14 | (0.14 – 0.15) | <0.001 | 0.71 | (0.71 – 0.72) | <0.001 | 0.64 | (0.64 – 0.65) | <0,001 |
| Diabetes without Complications | 0.11 | (0.11 – 0.12) | <0.001 | 0.89 | (0.89 – 0.89) | <0.001 | 0.80 | (0.80 – 0.80) | <0,001 |
| Diabetes with Complications | 0.20 | (0.2 – 0.2) | <0.001 | 0.82 | (0.82 – 0.82) | <0.001 | 0.70 | (0.70 – 0.70) | <0,001 |
| Paraplegia or Hemiplegia | 0.46 | (0.46 – 0.46) | <0.001 | 0.60 | (0.60 – 0.61) | <0.001 | 0.46 | (0.46 – 0.46) | <0,001 |
| Renal Disease | 0.17 | (0.17 – 0.17) | <0.001 | 0.84 | (0.84 – 0.84) | <0.001 | 0.69 | (0.69 – 0.70) | <0,001 |
| Cancer | 0.27 | (0.27 – 0.28) | <0.001 | 0.78 | (0.77 – 0.78) | <0.001 | 0.59 | (0.59 – 0.59) | <0,001 |
| Metastatic Cancer | 0.61 | (0.61 – 0.61) | <0.001 | 0.54 | (0.54 – 0.54) | <0.001 | 0.26 | (0.26 – 0.26) | <0,001 |
| AIDS/HIV | 0.31 | (0.3 – 0.33) | <0.001 | 0.66 | (0.64 – 0.67) | <0.001 | 0.61 | (0.58 – 0.63) | <0,001 |
| High-Risk Surgery |  |  |  |  |  |  |  |  |  |
| Intracranial | 0.74 | (0.74 – 0.74) | <0.001 | 0.43 | (0.43 – 0.43) | <0.001 | 0.18 | (0.18 – 0.18) | <0,001 |
| Thoracic | 0.24 | (0.24 – 0.24) | <0.001 | 0.72 | (0.72 – 0.72) | <0.001 | 0.62 | (0.61 – 0.62) | <0,001 |
| Abdominal | 0.06 | (0.06 – 0.06) | <0.001 | 0.93 | (0.93 – 0.93) | <0.001 | 0.91 | (0.91 – 0.91) | <0,001 |
| Cardiac Surgery | 0.12 | (0.12 – 0.12) | <0.001 | 0.85 | (0.84 – 0.85) | <0.001 | 0.83 | (0.83 – 0.84) | <0,001 |
| Transplantation Surgery | 1.41 | (1.4 – 1.43) | <0.001 | 0.36 | (0.36 – 0.36) | <0.001 | 0.09 | (0.09 – 0.09) | <0,001 |
| Number of Perioperative Organ Injuries |  |  |  |  |  |  |  |  |  |
| Delirium | 0.71 | (0.71 – 0.71) | <0.001 | 0.56 | (0.56 – 0.56) | <0.001 | 0.25 | (0.25 – 0.25) | <0.001 |
| Stroke | 0.42 | (0.42 – 0.43) | <0.001 | 0.64 | (0.64 – 0.65) | <0.001 | 0.35 | (0.34 – 0.35) | <0.001 |
| Acute Myocardial Infarction | 0.42 | (0.41 – 0.42) | <0.001 | 0.59 | (0.58 – 0.59) | <0.001 | 0.29 | (0.29 – 0.3) | <0.001 |
| Acute Respiratory Distress Syndrome | 0.61 | (0.61 – 0.62) | <0.001 | 0.42 | (0.42 – 0.42) | <0.001 | 0.17 | (0.16 – 0.17) | <0.001 |
| Pulmonary Embolism | 0.72 | (0.71 – 0.73) | <0.001 | 0.29 | (0.29 – 0.29) | <0.001 | 0.08 | (0.08 – 0.09) | <0.001 |
| Liver Injury | 0.03 | (0.02 – 0.04) | <0.001 | 0.20 | (0.20 – 0.20) | <0.001 | 0.05 | (0.05 – 0.05) | <0.001 |
| Acute Kidney Injury | 0.65 | (0.64 – 0.65) | <0.001 | 0.41 | (0.41 – 0.41) | <0.001 | 0.15 | (0.15 – 0.15) | <0.001 |
| Constant^g^ | 0.73 | (0.72 – 0.73) | <0.001 | --- | --- | --- | --- | --- | --- |

On the left, a linear regression model estimating the association with the logarithm of HLOS. In the middle, a competing risk model estimating the subdistribution hazard ratio for hospital discharge with death as competing risk. On the right, a proportional odds model estimating the odds ratio hospital-free of 90 days.

^a^a total of 393,157 in-hospital deaths were counted as competing risk; ^b^hospital-free days out of 90; ^c^n/a for proportional hazard and proportional odds models.

AIDS/HIV: Acquired Immune Deficiency Syndrome, Humane Immunodeficiency Virus; SHR: subdistribution hazard ratio; OR: odds ratio.

**Table S11: Risk factors of perioperative organ injury. Eight models in 28.350.953 patients describing the association of comorbidities and high-risk surgery with different types of perioperative organ injury.**

|  | Any organ injury | | Delirium | | | | Stroke | | | | AMI | | | | PE | | | | | ARDS | | | | | Liver Injury | | | | | | AKI | | | |  |
| --- | --- | --- | --- | --- | --- | --- | --- | --- | --- | --- | --- | --- | --- | --- | --- | --- | --- | --- | --- | --- | --- | --- | --- | --- | --- | --- | --- | --- | --- | --- | --- | --- | --- | --- | --- |
|  | OR | (95% CI) | OR | | (95% CI) | | OR | | (95% CI) | | OR | | (95% CI) | OR | | | (95% CI) | | OR | | | (95% CI) | | OR | | | | (95% CI) | | OR | | | (95% CI) |  |  |
| Age | 1.04 | (1.04 – 1.04) | | 1.05 | | (1.05 – 1.05) | | 1.03 | | (1.03 – 1.03) | | 1.02 | (1.02 – 1.02) | | | 1.02 | | (1.02 – 1.02) | | | 0.99 | | (0.99 – 0.99) | | | 1.00 | (1.00 – 1.00) | | 1.03 | | | (1.03 – 1.03) | | | |
| Sex | 0.73 | (0.72 – 0.73) | | 0.65 | | (0.65 – 0.66) | | 0.91 | | (0.90 – 0.92) | | 0.61 | (0.60 – 0.62) | | | 1.02 | | (1.00 – 1.03) | | | 0.57 | | (0.55 – 0.58) | | | 0.86 | (0.84 – 0.88) | | 0.73 | | | (0.72 – 0.73) | | | |
| Emergency hospital admission | 2.40 | (2.39 – 2.41) | | 1.93 | | (1.92 – 1.95) | | 2.14 | | (2.12 – 2.17) | | 2.20 | (2.18 – 2.23) | | | 2.02 | | (1.99 – 2.05) | | | 2.01 | | (1.97 – 2.06) | | | 1.83 | (1.79 – 1.87) | | 2.42 | | | (2.41 – 2.43) | | | |
| Charlson Comorbidity Score Items |  |  | |  | |  | |  | |  | |  |  | | |  | |  | | |  | |  | | |  |  | |  | | |  | | | |
| Myocardial Infarction | 0.93 | (0.92 – 0.94) | | 0.96 | | (0.95 – 0.97) | | 0.85 | | (0.83 – 0.88) | | 1.07 | (1.05 – 1.09) | | | 0.79 | | (0.76 – 0.82) | | | 0.73 | | (0.68 – 0.77) | | | 0.85 | (0.80 – 0.89) | | 0.89 | | | (0.88 – 0.9) | | | |
| Chronic Heart Failure | 2.68 | (2.67 – 2.69) | | 2.08 | | (2.06 – 2.09) | | 1.11 | | (1.10 – 1.13) | | 3.83 | (3.79 – 3.88) | | | 2.87 | | (2.81 – 2.92) | | | 4.27 | | (4.15 – 4.4) | | | 3.88 | (3.78 – 3.98) | | 3.16 | | | (3.14 – 3.18) | | | |
| Peripheral Vascular Disease | 1.45 | (1.44 – 1.46) | | 1.50 | | (1.49 – 1.52) | | 1.29 | | (1.27 – 1.31) | | 1.71 | (1.69 – 1.74) | | | 1.03 | | (1.01 – 1.06) | | | 1.24 | | (1.20 – 1.28) | | | 1.76 | (1.72 – 1.81) | | 1.39 | | | (1.38 – 1.40) | | | |
| Cerebrovascular Vascular Diesasee | 1.43 | (1.42 – 1.44) | | 1.50 | | (1.49 – 1.52) | | 1.58 | | (1.56 – 1.60) | | 1.34 | (1.31 – 1.36) | | | 1.01 | | (0.98 – 1.05) | | | 1.39 | | (1.32 – 1.45) | | | 1.21 | (1.16 – 1.27) | | 1.14 | | | (1.13 – 1.15) | | | |
| Dementia | 1.99 | (1.98 – 2.01) | | 3.38 | | (3.35 – 3.41) | | 0.85 | | (0.83 – 0.87) | | 0.83 | (0.81 – 0.85) | | | 0.98 | | (0.95 – 1.02) | | | 0.57 | | (0.53 – 0.62) | | | 0.75 | (0.70 – 0.80) | | 1.22 | | | (1.20 – 1.23) | | | |
| Chronic Pulmonary Disease | 1.32 | (1.31 – 1.32) | | 1.38 | | (1.37 – 1.39) | | 1.00 | | (0.98 – 1.02) | | 1.07 | (1.06 – 1.09) | | | 1.49 | | (1.46 – 1.52) | | | 1.96 | | (1.90 – 2.02) | | | 1.16 | (1.12 – 1.19) | | 1.33 | | | (1.32 – 1.35) | | | |
| Rheumatic Disease | 1.24 | (1.22 – 1.26) | | 1.15 | | (1.13 – 1.18) | | 1.10 | | (1.06 – 1.16) | | 1.13 | (1.09 – 1.18) | | | 1.57 | | (1.49 – 1.65) | | | 1.72 | | (1.58 – 1.87) | | | 1.32 | (1.21 – 1.43) | | 1.27 | | | (1.25 – 1.30) | | | |
| Peptic Ulcer Disease | 2.12 | (2.10 – 2.14) | | 1.65 | | (1.62 – 1.68) | | 1.28 | | (1.23 – 1.32) | | 2.31 | (2.24 – 2.37) | | | 1.61 | | (1.55 – 1.68) | | | 1.83 | | (1.73 – 1.93) | | | 1.94 | (1.86 – 2.02) | | 2.17 | | | (2.14 – 2.2) | | | |
| Mild Liver Disease | 1.88 | (1.86 – 1.90) | | 1.54 | | (1.51 – 1.57) | | 0.98 | | (0.95 – 1.02) | | 1.01 | (0.98 – 1.04) | | | 1.59 | | (1.53 – 1.65) | | | 2.70 | | (2.59 – 2.83) | | | 5.74 | (5.56 – 5.91) | | 2.22 | | | (2.19 – 2.25) | | | |
| Moderate to Severe Liver Disease | 3.25 | (3.20 – 3.30) | | 1.59 | | (1.54 – 1.64) | | 0.69 | | (0.65 – 0.74) | | 0.91 | (0.86 – 0.96) | | | 1.14 | | (1.06 – 1.22) | | | 3.26 | | (3.07 – 3.47) | | | 14.22 | (13.75 – 14.7) | | 4.58 | | | (4.50 – 4.66) | | | |
| Diabetes without complications | 1.28 | (1.27 – 1.28) | | 1.17 | | (1.16 – 1.18) | | 1.31 | | (1.30 – 1.33) | | 1.52 | (1.50 – 1.54) | | | 1.08 | | (1.06 – 1.10) | | | 1.43 | | (1.39 – 1.48) | | | 1.22 | (1.19 – 1.25) | | 1.31 | | | (1.30 – 1.31) | | | |
| Diabetes with complications | 1.24 | (1.23 – 1.25) | | 1.05 | | (1.04 – 1.07) | | 1.04 | | (1.02 – 1.07) | | 1.32 | (1.30 – 1.35) | | | 0.82 | | (0.79 – 0.85) | | | 0.85 | | (0.81 – 0.90) | | | 0.95 | (0.91 – 0.99) | | 1.41 | | | (1.40 – 1.43) | | | |
| Paraplegia or Hemiplegia | 4.64 | (4.60 – 4.68) | | 1.63 | | (1.60 – 1.65) | | 46.9 | | (46.3 – 47.5) | | 1.53 | (1.49 – 1.56) | | | 1.93 | | (1.86 – 2.00) | | | 2.15 | | (2.05 – 2.26) | | | 1.28 | (1.21 – 1.35) | | 1.60 | | | (1.58 – 1.62) | | | |
| Renal Disease | 1.75 | (1.74 – 1.76) | | 1.37 | | (1.36 – 1.38) | | 0.89 | | (0.88 – 0.90) | | 1.05 | (1.04 – 1.06) | | | 1.00 | | (0.98 – 1.02) | | | 0.97 | | (0.93 – 1.00) | | | 1.28 | (1.25 – 1.32) | | 2.51 | | | (2.49 – 2.53) | | | |
| Cancer | 1.32 | (1.31 – 1.33) | | 1.16 | | (1.15 – 1.18) | | 0.56 | | (0.55 – 0.58) | | 0.81 | (0.79 – 0.83) | | | 2.04 | | (2.00 – 2.09) | | | 1.39 | | (1.34 – 1.44) | | | 1.70 | (1.65 – 1.75) | | 1.61 | | | (1.59 – 1.62) | | | |
| Metastatic Cancer | 1.82 | (1.80 – 1.83) | | 1.56 | | (1.53 – 1.58) | | 0.49 | | (0.47 – 0.50) | | 0.73 | (0.71 – 0.76) | | | 4.33 | | (4.23 – 4.42) | | | 1.16 | | (1.10 – 1.21) | | | 2.97 | (2.87 – 3.07) | | 2.09 | | | (2.07 – 2.11) | | | |
| AIDS/HIV | 3.08 | (2.86 – 3.32) | | 3.11 | | (2.73 – 3.53) | | 1.08 | | (0.84 – 1.39) | | 1.49 | (1.16 – 1.91) | | | 2.33 | | (1.81 – 3.00) | | | 8.50 | | (7.19 – 10.05) | | | 4.22 | (3.41 – 5.21) | | 3.42 | | | (3.11 – 3.75) | | | |
| High-Risk Surgery |  |  | |  | |  | |  | |  | |  |  | | |  | |  | | |  | |  | | |  |  | |  | | |  | | | |
| Intracranial | 3.52 | (3.48 – 3.56) | | 4.43 | | (4.35 – 4.5) | | 3.45 | | (3.39 – 3.52) | | 1.35 | (1.29 – 1.42) | | | 3.68 | | (3.54 – 3.84) | | | 4.48 | | (4.22 – 4.75) | | | 2.87 | (2.66 – 3.11) | | 1.54 | | | (1.50 – 1.58) | | | |
| Thoracic | 2.34 | (2.32 – 2.35) | | 1.77 | | (1.75 – 1.78) | | 3.17 | | (3.12 – 3.23) | | 2.23 | (2.19 – 2.27) | | | 2.37 | | (2.32 – 2.42) | | | 3.58 | | (3.47 – 3.70) | | | 3.08 | (3.00 – 3.17) | | 2.29 | | | (2.27 – 2.30) | | | |
| Abdominal | 1.81 | (1.80 – 1.82) | | 1.34 | | (1.33 – 1.35) | | 1.96 | | (1.94 – 1.99) | | 1.42 | (1.40 – 1.44) | | | 2.10 | | (2.06 – 2.13) | | | 2.60 | | (2.54 – 2.67) | | | 3.93 | (3.83 – 4.02) | | 2.09 | | | (2.08 – 2.10) | | | |
| Cardiac Surgery | 3.83 | (3.81 – 3.85) | | 2.63 | | (2.61 – 2.66) | | 7.18 | | (7.06 – 7.30) | | 10.67 | (10.5 – 10.8) | | | 1.21 | | (1.16 – 1.25) | | | 2.41 | | (2.31 – 2.52) | | | 4.49 | (4.33 – 4.66) | | 1.95 | | | (1.93 – 1.97) | | | |
| Transplantation Surgery | 4.12 | (3.94 – 4.31) | | 6.15 | | (5.75 – 6.57) | | 1.93 | | (1.62 – 2.30) | | 0.87 | (0.74 – 1.02) | | | 2.84 | | (2.39 – 3.38) | | | 3.19 | | (2.74 – 3.70) | | | 6.58 | (6.03 – 7.17) | | 3.48 | | | (3.30 – 3.67) | | | |
| Constant | 0.00 | (0.00 – 0.00) | | 0.00 | | (0.00 – 0.00) | | 0.00 | | (0.00 – 0.00) | | 0.00 | (0.00 – 0.00) | | | 0.00 | | (0.00 – 0.00) | | | 0.00 | | (0.00 – 0.00) | | | 0.00 | (0.00 – 0.00) | | 0.00 | | | (0 – 0) | | | |

Regression model analysis identified common and specific risk factors for different organ injuries. Transplant surgery, a history of MI, and liver disease are the three leading risk factors for developing any organ injury.

AIDS/HIV: Acquired Immune Deficiency Syndrome. Human Immunodeficiency Virus; HR: hazard ratio; OR: odds ratio.

**Table S12:Taxonomy of the *Operationen- und Prozedurenschlüssel* (OPS), the German version of the *International Classification of Procedures in Medicine* (ICPM), showing in particular details of the subsections from “Chapter 5 – Operations”.**

| Chapter 1 | Diagnostic Measures | |
| --- | --- | --- |
| Chapter 3 | Diagnostic Imaging | |
| Chapter 5 | Operations | |
|  | 5-01 to 5-05 | Operations on the Nervous System |
|  | 5-06 to 5-07 | Operations on Endocrine System |
|  | 5-08 to 5-16 | Operations on the Eyes |
|  | 5-18 to 5-20 | Operations on the Ears |
|  | 5-21 to 5-22 | Operations on Nose, Mouth and Pharynx |
|  | 5-23 to 5-28 | Operations in the Oral cavity and Face |
|  | 5-29 to 5-31 | Operations on Pharynx, Larynx and Trachea |
|  | 5-32 to 5-34 | Operations on the Lungs and Bronchi |
|  | 5-35 to 5-37 | Operations on the Heart |
|  | 5-38 to 5-39 | Operations on Blood Vessels |
|  | 5-40 to 5-41 | Operations on the Hemopoietic and Lymphatic Systems |
|  | 5-42 to 5-54 | Operations on the Digestive System |
|  | 5-55 to 5-59 | Operations on the Urinary Tract |
|  | 5-60 to 5-64 | Operations on Male Genital Organs |
|  | 5-65 to 5-71 | Operations on Female Genital Organs |
|  | 5-72 to 5-75 | Obstetric Operations |
|  | 5-76 to 5-77 | Maxillofacial Operations |
|  | 5-78 to 5-86 | Operations on the Musculoskeletal System |
|  | 5-87 to 5-88 | Operations on the Breasts |
|  | 5-89 to 5-92 | Operations on Skin and Subcutaneous Tissue |
|  | 5-93 to 5-99 | Additional Information on Operations |
| Chapter 6 | Medications | |
| Chapter 8 | Non-Surgical Therapeutic Measures | |
| Chapter 9 | Complimentary Measures | |

**Table S13: Details of variable transcoding for procedures and diagnoses.**

| **Organ Injuries**^a^ | |
| --- | --- |
| Delirium | F05 |
| Stroke | I63, I64 |
| Acute Myocardial Infarction | I21, I22 |
| Adult Respiratory Distress Syndrome | J80 |
| Pulmonary Embolism: | I26 |
| Liver Injury | K7040, K7042, K712, K720, K7210 |
| Acute Kidney Injury | N17 |
| **Comorbidities**^a^ | |
| Acute Myocardial Infarction | I252 |
| Congestive Heart Failure | I43, I50, I099, I110, I130, I132, I255, I420, I425, I426, I427, I428, I429, P290 |
| Peripheral Vascular Disease | I70, I71, I731, I738, I739, I771, I790, I792, K551, K558, K559, Z958, Z959, |
| Cerebrovascular Disease | G45, G46, I60, I61, I62, I65, I66, I67, I68, I69, H340 |
| Dementia | F00, F01, F02, F03, G30, G311 |
| Chronic Pulmonary Disease | J40, J41, J42, J43, J44, J45, J46, J47, J60, J61, J62, J63, J64, J65, J66, J67, I278, I279, J684, J701, J703 |
| Rheumatologic Disease | M05, M32, M33, M34, M06, M315, M351, M353, M360 |
| Peptic Ulcer Disease | K25, K26, K27, K28, |
| Mild Liver Disease | B18, K73, K74, K700, K701, K702, K703, K709, K713, K714, K715, K717, K760, K762, K763, K764, K768, K769, Z944 |
| Moderate/Severe Liver Disease | K704 (excluding K7040 and K7042), K711, K721, K729, K765, K766, K767, I850, I859, I864, I982 |
| Diabetes without complications | E100, E101, E106, E108, E109, E110, E111, E116, E118, E119, E120, E121, E126, E128, E129,E130, E131, E136, E138, E139, E140, E141, E146, E148, E149 |
| Diabetes with chronic complications | E102, E103, E104, E105, E107, E112, E113 , E114, E115, E117, E122, E123, E124, E125, E127,E132, E133, E134, E135, E137, E142, E143, E144, E145, E147 |
| Hemiplegia or Paraplegia | G81, G82, G041, G114, G801, G802, G830, G831, G832, G833, G834, G839 |
| Renal Disease | N18, N19, N052, N053, N054, N055, N056, N057, N250, I120, I131, N032, N033, N034, N035, N036, N037, Z490, Z491, Z492, Z940, Z992 |
| Cancer | C00, C01, C02, C03, C04, C05, C06, C07, C08, C09, C10, C11, C12, C13, C14, C15, C16, C17, C18, C19, C20, C21, C22, C23, C24, C25, C26, C30, C31, C32, C33, C34, C37, C38, C39, C40, C41, C43, C45, C46, C47, C48, C49, C50, C51, C52, C53, C54, C55, C56, C57, C58, C60, C61, C62, C63, C64, C65, C66, C67, C68, C69, C70, C71, C72, C73, C74, C75, C76, C81, C82, C83, C84, C85, C88, C90, C91, C92, C93, C94, C95, C96, C97 |
| Metastatic Cancer | C77, C78, C79, C80 |
| AIDS/HIV | B20, B21, B22, B24 |
| **High-Risk Surgery**^b^ | |
| Intracranial Surgery | 501, 502, 5075, 5076, |
| Thoracic Surgery^c^ | 532, 533, 534, 539, 542, 5077, 5078, 5314, 5316, 5387, 5405, 53840, 53841, 53843, 53844, 5384d, 5384e, 5384f, 538a7, 538aa, 53804, 53814, 53834, 53884, 53894, 53954, 53964, 53974, 538030, 538031, 538032, 538130, 538131, 538132, 538230, 538231, 538232, 538330, 538331, 538332, 538830, 538831, 538832, 538390, 538931, 538932, 539530, 539531, 539532, 539630, 539631, 539632, 539730, 539731, 539732 |
| Cardiac Surgery | 535, 536, 537 |
| Abdominal Surgery | 543, 544, 545, 546, 547, 550, 551, 552, 553, 554, 560, 565, 566, 568, 569, 5070, 5071, 5072, 5073, 5412, 5413, 5419, 53844, 53845, 53846, 53847, 53805, 53815, 53825, 53835, 53885, 53895, 53806, 53816, 53826, 53836, 53886, 53896, 53955, 53965, 53975, 53956, 53966, 53976 |
| Transplantation Surgery | 5528, 5375, 5335, 5504, 5555 |

^a^ICD-10-GM codes, ^b^OPS codes, ^c^without cardiac surgery.

AIDS: acquired immune deficiency syndrome, HIV: Human immunodeficiency virus.

Diagnoses (ICD-10-GM) and procedure codes (OPS) for every newly transcoded variable of exposures and confounders. No wildcard symbols are presented in this table for better readability; codes in this table represent all hierarchal subcodes beginning with the same digi

# Supplementary References

1. Internationale statistische Klassifikation der Krankheiten und verwandter Gesundheitsprobleme. 2020. (Accessed 23 June, 2020, at h<ttps://www.dimdi.de/static/de/klassifikationen/icd/icd-10-gm/kode-suche/htmlgm2020/.>)

2. OPS. 2020. at <https://www.dimdi.de/dynamic/en/classifications/ops>.)

3. Born S, Dame C, Matthäus-Krämer C, et al. Epidemiology of Sepsis Among Children and Neonates in Germany: Results From an Observational Study Based on Nationwide Diagnosis-Related Groups Data Between 2010 and 2016. Crit Care Med 2021.

4. Charlson ME, Pompei P, Ales KL, MacKenzie CR. A new method of classifying prognostic comorbidity in longitudinal studies: development and validation. J Chronic Dis 1987;40:373-83.

5. Quan H, Sundararajan V, Halfon P, et al. Coding algorithms for defining comorbidities in ICD-9-CM and ICD-10 administrative data. Med Care 2005;43:1130-9.

6. Luque-Fernandez MA, Maringe C, Nelson P. CVAUROC: Stata module to compute Cross-validated Area Under the Curve for ROC Analysis after Predictive Modelling for Binary Outcomes. 2017.

7. Daniels B. CROSSFOLD: Stata module to perform k-fold cross-validation. 2012.
